# A reproducible ultrasound localization microscopy framework for the quantitative imaging of hepatocellular carcinoma on a clinical ultrasound system

**DOI:** 10.7150/thno.133561

**Published:** 2026-07-20

**Authors:** Jiajia Tang, Huazhen Liu, Baoluhe Zhang, Shunda Du, Long Zou, Jinghui Liang, Meng Yang

**Affiliations:** 1 Department of Ultrasound, Peking Union Medical College Hospital, Chinese Academy of Medical Sciences & Peking Union Medical College, Beijing 100730, China.; 2 Department of Liver Surgery, State Key Laboratory of Common Mechanism Research for Major Diseases, Peking Union Medical College Hospital, Chinese Academy of Medical Sciences & Peking Union Medical College, Beijing 100730, China.; 3 Department of Pathology, Peking Union Medical College Hospital, Chinese Academy of Medical Sciences & Peking Union Medical College, Beijing 100730, China.

**Keywords:** ultrasound localization microscopy (ULM), reproducible framework, hepatocellular carcinoma, quantitative parameters, microvascular assessment

## Abstract

**Rationale:**

Poor differentiation and microvascular invasion (MVI) are crucial prognostic indicators for hepatocellular carcinoma (HCC), but evaluating them before surgery is still difficult. Our goal was to develop a reproducible framework for ultrasound localization microscopy (ULM) using a clinical ultrasound system and assess its effectiveness in predicting tumor differentiation and MVI.

**Methods:**

Participants were prospectively enrolled and underwent contrast-enhanced ultrasound and ULM imaging. We first compared frame selection strategies by calculating coefficients of variation (COVs) of ULM parameters derived from motion-variance curve (MVC) alone versus MVC combined with time-intensity curve (TIC). ULM resolution and parameter stability were then assessed across low (0–399), medium (400–699), and high (700–1000) frame counts. Regions of interest (ROIs) were manually drawn on grayscale ultrasound and mapped to ULM images. Inter-operator agreement was evaluated using intraclass correlation coefficients (ICCs). Participants were grouped by pathological differentiation and MVI status. The predictive performance of ULM parameters was assessed with multivariable logistic regression.

**Results:**

Sixty-one HCC participants were enrolled (11 poorly differentiated, 50 well differentiated; 30 MVI-positive, 31 MVI-negative). The MVC+TIC strategy yielded significantly lower COVs, indicating higher repeatability of ULM parameters. Medium frame counts (400–699) provided high resolution (minimum vessel diameter 91.3 ± 22.7 μm) and stable parameters (COV < 20%), and were therefore selected for ULM reconstruction. All ULM parameters showed high inter-operator agreement (ICC 0.876–0.988). Based on the established ULM framework, higher intratumoral mean curvature was independently associated with poor differentiation [area under the curve (AUC) 0.91], and higher peritumoral mean curvature independently predicted MVI status (AUC 0.78).

**Conclusion:**

The reproducible ULM framework enables stable, high-resolution microvascular imaging of HCC. ULM-derived parameters hold potential as novel biomarkers for predicting differentiation grade and MVI status of HCC.

## Introduction

Primary liver cancer imposes a substantial global health burden and hepatocellular carcinoma (HCC) accounts for 75-85% of primary liver cancer 1,2. Despite advancements in surveillance and treatment strategies, the prognosis of HCC remains unsatisfactory largely due to the high recurrence rate 3. Pathological tumor grade and microvascular invasion (MVI) stand as the most validated prognostic indicators 4,5. However, these characteristics can only be determined by pathological examination after surgical resection. It is crucial to employ non-invasive approaches to anticipate recurrence-related characteristics.

Earlier studies have developed diagnostic models using preoperative imaging techniques to predict high-recurrence HCC 6,7. Ultrasound is a key imaging technique for assessing liver nodules, providing real-time imaging and being widely accessible. Contrast-enhanced ultrasound (CEUS) allows for a more detailed analysis of tumor blood flow patterns. The first application of CEUS to liver tumors was reported in 1986 8. Subsequent advances included early transpulmonary agents such as Albunex® 9, more stable clinical formulations like Levovist® 10, and second-generation microbubbles with harmonic imaging 11. These key improvements have established CEUS as a clinical standard for liver perfusion assessment 12-14. Nevertheless, CEUS resolution remains fundamentally constrained by the acoustic diffraction limit, precluding detailed visualization and quantification of the tumor microvasculature. HCC exhibits a highly heterogeneous microvascular network, predominantly composed of small-diameter (< 200 µm) and low-flow (< 10 mm/s) vessels 15,16. A high-resolution imaging technique is required to more meticulously delineate the microcirculatory features of HCC.

Ultrasound localization microscopy (ULM) is a new super-resolution imaging method. Its core principle is to localize single microbubbles across a series of frames and reconstruct the microvascular network. Unlike real-time imaging, ULM reconstruction is performed as a post-processing step after CEUS acquisition 17-19. The accumulated localizations allow for quantitative multiparametric characterization of microvascular hemodynamics. ULM has been successfully demonstrated in preclinical models and clinical pilot studies of neurological, cardiovascular, and tumor diseases 20-22.

Several clinical investigations have specifically extended ULM to liver imaging. Lok et al. employed ULM in human livers and achieved a microvessel resolution of approximately 170 μm 23. Huang et al. performed high-frame-rate ULM in healthy and diseased livers, and reported a resolvable vessel width of 153 μm in the normal liver 24. These studies, however, used linear-array probes with an imaging depth generally limited to less than 5 cm, which is insufficient for deep hepatic lesions commonly encountered in clinical practice. Zeng et al. addressed the depth limitation by applying ULM to focal liver lesions with a convex-array probe and diverging-wave ultrafast imaging, achieving a minimum vessel diameter of 128.4 ± 18.6 μm at depths up to 8.6 cm 25. Nonetheless, their reconstruction workflow remained off-line, which constrains integration into routine clinical practice. A recent study integrated CEUS with ULM on a clinical ultrasound system, enabling simultaneous CEUS acquisition and on-system ULM reconstruction. This study successfully detected microvessels as small as 60 μm in small HCC lesions and further demonstrated associations between quantitative ULM parameters and HCC histological features 26.

Despite these improvements, there are various challenges to the extensive application of ULM in HCC. Initially, different imaging planes and acquisition times produce diverse raw data. In addition, non-uniform frame selection across different studies results in unstable image resolution and undermines the consistency of quantitative parameters. For the same ULM image, different methods of ROI delineation by observers lead to quantitative ULM values that are not reproducible. Thus, the objective of this study was to address these issues by creating a reproducible ULM framework for HCC with a clinical ultrasound system. We aimed to determine a fixed time window for acquiring echo signals, optimize the strategy for frame selection, and examine the variability in quantitative parameters among operators. Furthermore, we sought to explore the value of the ULM parameters for revealing microvascular phenotypes associated with differentiation grade and MVI status.

## Methods

### Participants

This prospective single-center study was approved by the ethics committee of Peking Union Medical College Hospital (I-24PJ1499). All participants provided written informed consent. The study protocol was registered at ClinicalTrials.gov (NCT07101237). Participants were consecutively enrolled between January 2025 and October 2025. The inclusion criteria were as follows: (1) presence of a suspected malignant liver tumor; (2) no prior anticancer treatment before CEUS examination; (3) no history of other malignancies; (4) CEUS examination performed within one week prior to surgery. Exclusion criteria: (1) suboptimal image quality on CEUS or ULM; (2) incomplete clinical or pathological data. Demographic data and clinical characteristics were collected from each participant using the electronic medical record system.

### US technique and CEUS process

Grayscale US, CEUS, and ULM were performed using a clinical ultrasound system (ULTIMUS 9E, VINNO, Suzhou, China) equipped with a convex abdominal probe (S1–8C; frequency range 1.5–7 MHz; center frequency 3.2 MHz). For CEUS imaging, the contrast agent (SonoVue, Bracco, Milano, Italy) was prepared by adding 5 mL of sterile saline to the lyophilized powder, followed by vigorous hand agitation for 20 seconds to obtain a homogeneous microbubble suspension. The suspension contained sulfur hexafluoride microbubbles at a concentration of 8 µL/mL (approximately 1×10^8^ to 5×10^8^ microbubbles per mL) 27. A 1 mL bolus of suspension was administered intravenously, followed immediately by a 5 mL saline flush. CEUS was performed at a low mechanical index (0.056) to minimize microbubble destruction. Real-time dual-mode imaging (concurrent US and CEUS) was used to optimize image depth and gain. The CEUS process continued for a total of six minutes. The time-intensity curve (TIC) was generated from the whole CEUS process.

### ULM reconstruction

All ULM reconstruction steps were performed using the built-in ULM function of the VINNO Ultimus 9E ultrasound scanner. (1) Echo signal acquisition: a frame rate of 100 Hz was employed to capture microbubble echoes; (2) Microbubble signal separation: Singular value decomposition (SVD) with an adaptive threshold was applied to beamformed in-phase and quadrature (IQ) data to isolate microbubble signals from tissue clutter. The adaptive threshold was determined based on the similarity of spatial singular vectors, as proposed in a previous study 28. A windowed bandpass filter in the temporal frequency domain was applied to the SVD output. This additional step mitigated residual clutter near the temporal boundaries, thereby improving the robustness of microbubble separation under varying hemodynamic conditions; (3) Motion artifact suppression: gross motion was minimized through probe fixation and a 10-second breath-hold. Then an intelligent imaging-plane guidance system was used to maintain the target lesion within the field of view (**Note S1**). A motion-variance curve (MVC) was generated from frame-wise image correlation. According to MVC, only frames with variability less than 10% were retained (**Note S2**). Image-based optical flow method was utilized to address residual cardiac pulsation and heterogeneous tissue deformation. This method is built on the brightness constancy assumption and the Lucas-Kanade algorithm. It estimates a motion vector for each pixel through dense computation 26, 27. The resulting dense motion field could accurately capture complex local non-rigid deformation. This method was then used to warp each microbubble-dominant frame to a common reference coordinate system; (4) Microbubble localization: Iterative point-spread-function (PSF) deconvolution compensates for image distortion caused by the system’s point-spread function 31. It also contracts overlapping microbubble signals, thereby mitigating the localization challenge and facilitating separation at clinically used contrast doses. A gradient-optimized algorithm determines the centroid of each contracted microbubble signal; (5) Microbubble tracking: An accelerated Kalman tracking with linear motion constraints was implemented 32. The strength of this algorithm lies in its integration of historical velocity information and a maximum expected velocity constraint. Under a frame rate of 100 Hz, this approach helped achieve robust linking of microbubble trajectories, allowing velocities up to approximately 50 mm/s to be tracked. These features contributed to alleviating the challenges typically associated with tracking at low frame rates. (6) Trajectory mapping and quantitative analysis: Accumulated microbubble trajectories were used to reconstruct microvascular networks. ULM maps were generated and ULM quantitative parameters were calculated.

### ULM quantitative calculation

ULM resolution indices include vessel count, vessel diameter and inter-vessel distance. On the density map, a fitted density curve was extracted along a line perpendicular to the vessel orientation. Vessel count was the total number of peaks along that line. Vessel diameter was defined as the full width at half maximum of each density peak. Inter-vessel distance was the distance between adjacent peaks (see **Note S3** for calculation method and resolution analysis).

ULM structural-functional parameters encompass density parameters [maximum, minimum, mean, std (standard deviation) density and density entropy], velocity parameters (maximum, minimum, mean, std velocity and velocity entropy), curvature parameters (maximum, minimum, mean and std curvature), as well as hemodynamic and morphological parameters (vessel ratio, complexity level, blood volume, and perfusion index). For each ROI, summary statistics and relevant measures were computed using the built-in algorithms of the VINNO system (see **Note S4** for detailed formulas).

### Frame selection strategy for HCC ULM

TIC visually reflects the contrast wash-in pattern, allowing identification of the peak perfusion phase. Combining TIC with MVC defines a frame range that captures the peak perfusion status while excluding motion-contaminated frames. To confirm the improvement of the TIC+MVC frame selection strategy, we compared the repeatability of ULM parameters from two frame selection methods: (1) MVC group: frames selected only by the MVC; (2) TIC + MVC group: frames restricted to the peak-intensity window with motion variability ≤ 10%. For each parameter, the coefficients of variation (COV) were calculated from the three ULM values obtained under each strategy (MVC and MVC + TIC). The COV from the two strategies were then compared using the Wilcoxon signed-rank test. Two-sided *p* value < 0.05 was considered statistically significant.

To identify the appropriate frame-count interval for reconstruction, ULM images were generated across ten consecutive intervals: 0-99, 100–199, 200–299, 300–399, 400–499, 500–599, 600–699, 700–799, 800–899, and 900–1000 frames. For each interval, ULM resolution indices (vessel count, minimal vessel diameter, minimal inter-vessel distance) and structural-functional parameters (density, velocity, curvature, and hemodynamic and morphological parameters) were calculated. These parameters were plotted against frame-count intervals to visualize trends.

The ten intervals were grouped into low-frame (0–399), medium-frame (400–699), and high-frame (700–1000) groups. Comparisons of ULM parameters among the three frame groups were performed using repeated-measures ANOVA with Bonferroni post-hoc test when data were normally distributed. When the normality assumption was violated, the Friedman test was used, followed by Wilcoxon signed-rank tests with Bonferroni correction for pairwise comparisons.

To assess measurement consistency within a given frame group, COV of each ULM parameter was calculated. A COV below 20% was considered to indicate high consistency. As the low-frame group was deemed greatly inadequate, only the COVs of the medium-frame and high-frame groups were compared. Differences were assessed using the paired t-test for normally distributed differences or the Wilcoxon signed-rank test otherwise. A two-sided *p* value < 0.05 was considered statistically significant.

### Quantitative analysis of ULM parameters in HCC

Tumor boundaries were delineated manually on grayscale US based on the visible interface between the lesion and adjacent liver parenchyma. The intratumoral ROI comprised the area enclosed within the tumor boundary. The peritumoral ROI was defined as a 0.5 cm annular extension outward from the tumor boundary, excluding the intratumoral region. Selected ULM parameters were computed separately for the intratumoral and peritumoral regions.

Thirty cases were randomly selected for independent ROI delineation by two radiologists (J.J.T. and H.Z.L., both with 5 years of US experience). Sample size calculation was detailed in **Note S5**. Inter-observer agreement of ULM parameters was assessed using the intraclass correlation coefficient (ICC). ICC thresholds were defined as: < 0.5, poor; 0.5 – 0.75, moderate; 0.75 – 0.9, good; > 0.9, excellent. Agreement was further visualized with Bland–Altman plots.

### Validation of ULM parameters against CEUS-derived perfusion metrics

For CEUS analysis, ROIs were delineated on grayscale US using the same method as described for ULM parameter quantification. Quantitative CEUS metrics were derived from TIC generated within the intratumoral ROI, consisting of rising time, time to peak, peak intensity, mean transit time (MTT), and area under the curve (AUC). The association between CEUS metrics and ULM parameters was evaluated using correlation analysis. Depending on data distribution, Pearson's correlation coefficient (normally distributed data) or Spearman's rank correlation coefficient (non-normally distributed data) was applied. Correlation strength was interpreted as follows: < 0.30, weak; 0.30 – 0.50, moderate; 0.50 – 0.70, strong; > 0.70, very strong.

### Pathological examination

Resected tumors were analyzed pathologically with hematoxylin-eosin (H&E) staining and CD34 immunohistochemical staining. Pathological diagnoses were independently reviewed by two pathologists (more than 10 years of experience). HCC was diagnosed according to the World Health Organization classification. Pathological differentiation was graded according to the Edmondson-Steiner (ES) criteria: ES grade I–II was defined as well-differentiated HCC (w-HCC), and grade III–IV as poorly-differentiated HCC (p-HCC). MVI was identified as tumor cell nests within a portal vein, hepatic vein, or large capsular vessel of the adjacent hepatic parenchyma. MVI was graded as M0 (absent), M1 (≤ 5 tumor emboli or single vessel involvement), or M2 (> 5 emboli and/or involvement of ≥ 2 vessels). HCC cases were divided into MVI-positive (M1 and M2) and MVI-negative (M0) groups.

### Statistical analysis

Statistical analyses were performed using R software (version 4.1.1), GraphPad Prism (version 10), and IBM SPSS Statistics (version 27). The distribution of continuous variables was assessed using histograms and the Shapiro-Wilk test. Normally distributed data were presented as mean ± standard deviation (SD), and non-normally distributed data as median with interquartile range (IQR). For comparisons of continuous variables between two independent groups, the Student’s t-test or the Mann–Whitney U test was applied. Categorical variables were expressed as frequencies and percentages. Group comparisons for categorical data were performed using the chi-square test or Fisher’s exact test.

ULM parameters that exhibited statistically significant differences between groups were entered into univariate logistic regression models. Variables with a *p* value < 0.05 in univariate analysis were entered into multivariate logistic regression to identify independent predictors. Odds ratios (ORs) with 95% confidence intervals (CIs) were calculated. Predictive performance was evaluated using receiver operating characteristic (ROC) curve analysis. The corresponding sensitivity, specificity, accuracy, and area under the ROC curve (AUC) were computed. All statistical tests were two-sided, and a *p* value < 0.05 was considered statistically significant.

## Results

### Participant characteristics

**Figure 1:**
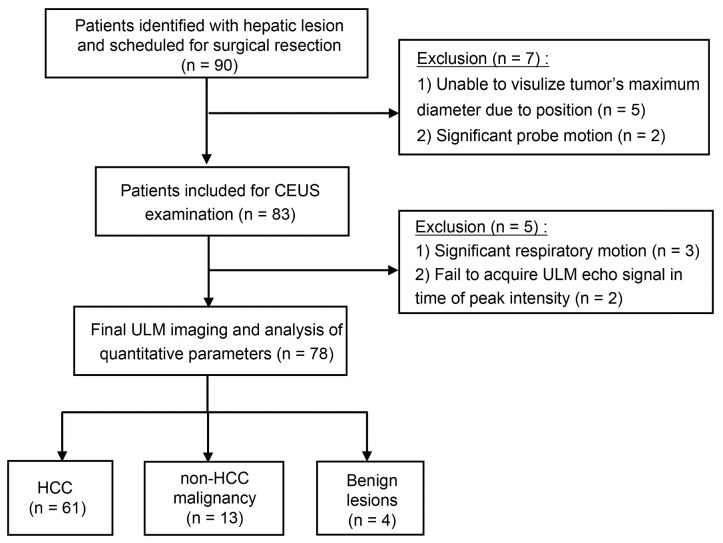
Participant enrollment flowchart. CEUS, contrast-enhanced ultrasound; ULM, ultrasound localization microscopy; HCC, hepatocellular carcinoma.

A total of 90 patients who underwent hepatectomy for suspected malignant liver tumors were initially considered for inclusion in this study (**Figure 1**). 78 patients ultimately underwent both CEUS and ULM. The final pathological diagnoses included 61 HCCs, 13 non-HCC malignant tumors, and 4 benign lesions. The non-HCC malignancies consisted of 8 intrahepatic cholangiocarcinomas (ICCs), 3 combined HCC-ICCs, 1 lymphoma, and 1 neuroendocrine carcinoma. The benign lesions comprised 2 cases of focal nodular hyperplasia (FNH), 1 hepatic adenoma, and 1 inflammatory pseudotumor.

Among the 61 HCC patients, the distribution of tumor differentiation grades was as follows: ES grade I in 2 patients (3.3%), grade II in 48 patients (78.7%), and grade III in 11 patients (18.0%). Based on the established criteria, 50 patients (82.0%) were classified as w-HCC and 11 patients (18.0%) as p-HCC. MVI was identified in 30 of the 61 HCC patients (49.2%), comprising 26 patients (42.6%) with MVI 1 and 4 patients (6.6%) with MVI 2. The remaining 31 patients (50.8%) were MVI-negative (MVI 0).

### Baseline CEUS and ULM characteristics

For the 61 HCCs finally included, the median depth was 5.1 cm (IQR, 4.15–6.15). CEUS metrics were calculated based on the TIC. The median arrival time was 13.31 seconds (IQR, 10.59–15.92). The median rising time was 17.02 seconds (IQR, 14.31–22.89). The median time to peak intensity was 31.95 seconds (IQR, 25.88–36.94), and MTT was 115.50 ± 29.50 seconds. For ULM imaging, the median number of reconstruction frames was 609 (IQR, 580–650), and the median reconstruction time was 42 seconds (IQR, 41–45). The minimum vessel diameter was 91.30 ± 22.70 μm (range: 55–150 μm) and the minimum inter-vessel distance was 168.00 ± 48.55 μm (range: 100–280 μm), both substantially smaller than the 240 μm diffraction limit (3.2 MHz).

### Improved repeatability of ULM parameters using TIC+MVC frame selection

**Figure 2:**
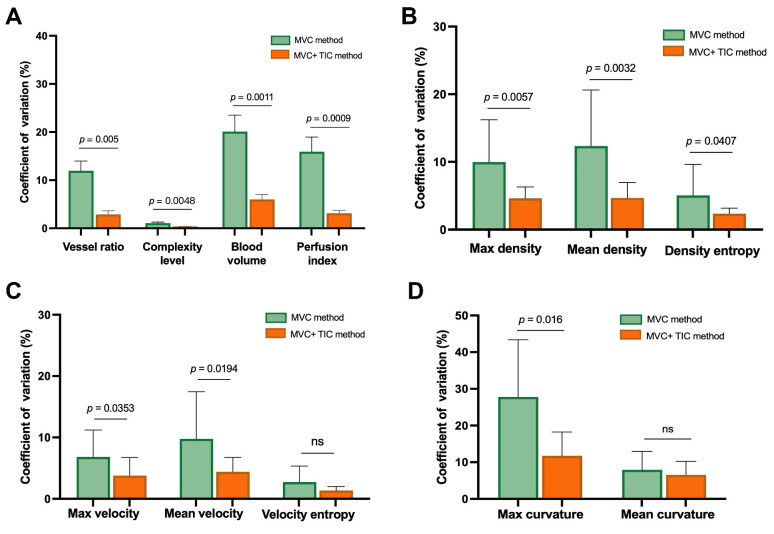
** Comparison of COVs between MVC and TIC+MVC frame selection strategies.** Bars represent mean COV for the MVC and TIC+MVC groups, with error bars showing standard deviation (n = 20). (A) hemodynamics and vascular morphology parameters, (B) density parameters, (C) velocity parameters, and (D) curvature parameters. The TIC+MVC method yielded significantly lower COV values for most parameters (*p* < 0.05). Two parameters exhibited numerically lower COV without statistical significance (ns, *p* ≥ 0.05). MVC, motion-variance curve; TIC, time-intensity curve; COV, coefficient of variation.

The combined TIC+MVC frame selection strategy yielded consistently lower COVs compared with the MVC-only approach across all evaluated ULM structural-functional parameters. Statistically significant reductions in COV were observed for vessel ratio, complexity level, blood volume, perfusion index, max density, mean density, density entropy, max velocity, mean velocity, and max curvature (all *p* < 0.05). A summary comparison of COV distributions between the two strategies is shown in** Figure 2**. Overall, the TIC+MVC strategy was associated with greatly lower COVs, indicating improved repeatability of ULM parameters.

### Appropriate frame-count interval for ULM reconstruction

**Figure 3:**
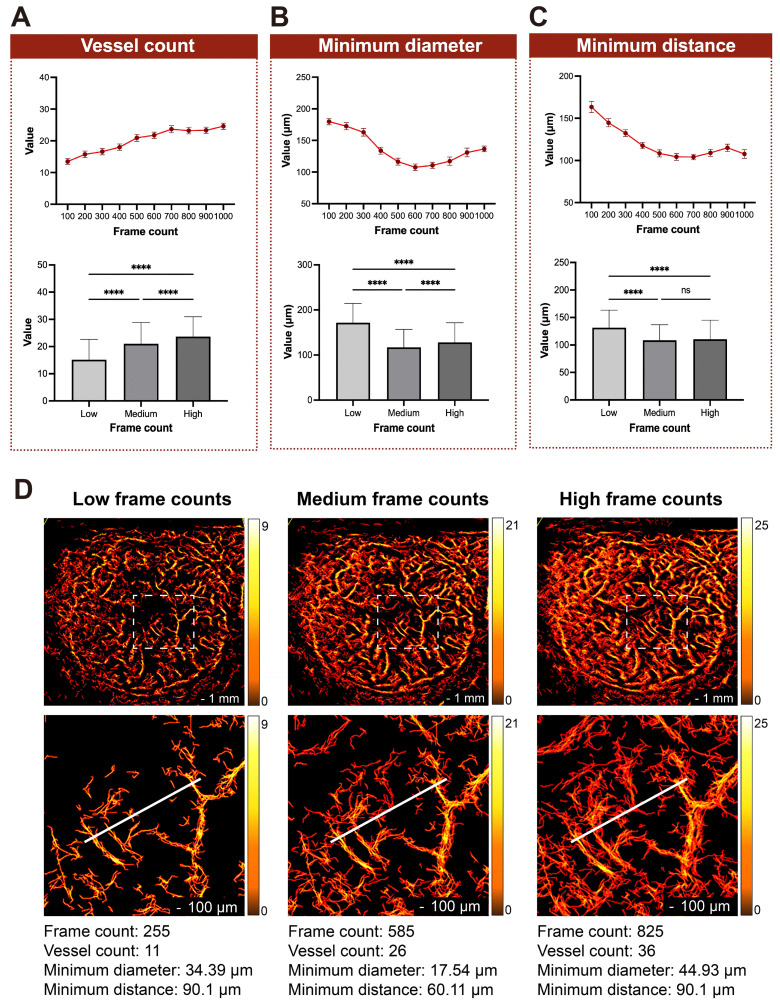
** ULM resolution indices across increasing frame counts.** Line plots depict the trajectory of (A) vessel count, (B) minimum vessel diameter, and (C) minimum inter-vessel distance across increasing frame counts. Embedded bar graphs display group-wise comparisons among low-frame (0–399), medium-frame (400–699), and high-frame (700–1000) groups. Data are presented as mean ± standard deviation (n = 61). **** *p* < 0.0001, ns: *p* ≥ 0.05; (D) Representative ULM images reconstructed at low (255), medium (585), and high (825) frame counts. Resolution indices measured along the path indicated by the white ruler are shown beneath each panel. ULM, ultrasound localization microscopy. Scale bar: 1 mm and 100 μm.

Changes of three ULM resolution indices were depicted by line plots. As the number of frames grew, vessel count rose progressively, with the growth rate slowing down beyond approximately 700 frames. In contrast, minimum vessel diameter and minimum inter-vessel distance both declined initially, reached their lowest values at around 600 frames, and then gradually increased. In group comparison analysis, all three resolution indices differed significantly across the low-frame (0-399), medium-frame (400-699), and high-frame (700-1000) groups (repeated-measures ANOVA, all *p* < 0.001). Vessel count peaked in the high-frame group and was significantly higher than in both the low-frame and medium-frame groups (all *p* < 0.05) (**Figure 3A**). Minimum diameter was lowest in the medium-frame group and differed significantly from both the low-frame and high-frame groups (all *p* < 0.05) (**Figure 3B**). Minimum distance was significantly lower in the medium-frame and high-frame groups compared with the low-frame group, with no significant difference observed between the medium and high-frame groups (**Figure 3C**). A representative case illustrating these resolution trends is shown in **Figure 3D**.

Curves and group comparisons for all ULM structural-functional parameters are summarized in **Note S6**. Hemodynamic parameters (vessel ratio, blood volume, perfusion index) and density parameters (maximum density, mean density, density entropy) differed significantly across the three frame groups (repeated-measures ANOVA, all *p* < 0.001). Post-hoc analyses revealed that these differences were primarily driven by the low-frame group, while no significant differences were detected between the medium and high-frame groups for any of the evaluated parameters. Velocity and curvature parameters showed no significant variation across frame counts (all *p* ≥ 0.05).

**Figure 4:**
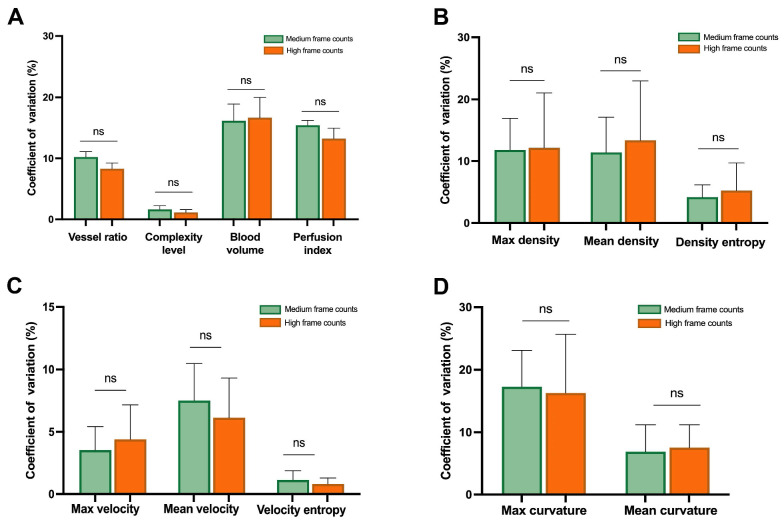
** Within-group repeatability of ULM structural-functional parameters across medium-frame and high-frame groups.** Bars represent the mean COV with standard deviation (n = 61). Parameters are categorized as (A) hemodynamic and morphological, (B) density, (C) velocity, and (D) curvature. No significant differences were observed between the two groups for any parameter category (ns, *p* ≥ 0.05). ULM, ultrasound localization microscopy; COV, coefficient of variation.

Given the absence of significant parameter differences between the medium-frame and high-frame groups, within-group repeatability was assessed via COV. Mean COV values remained below 20% for all parameters in both groups, indicating high measurement consistency (**Figure 4**). No significant difference in COV was observed between the medium-frame and high-frame groups (all *p* ≥ 0.05).

On the basis of the observed parameter stability and resolution performance, and considering the clinical requirement of patient breath-hold tolerance, the medium-frame interval (400-699 frames; approximately 4-7 seconds) was selected as the appropriate frame-count interval for subsequent ULM reconstruction.

### Inter-observer agreement of ULM structural-functional parameters

**Figure 5:**
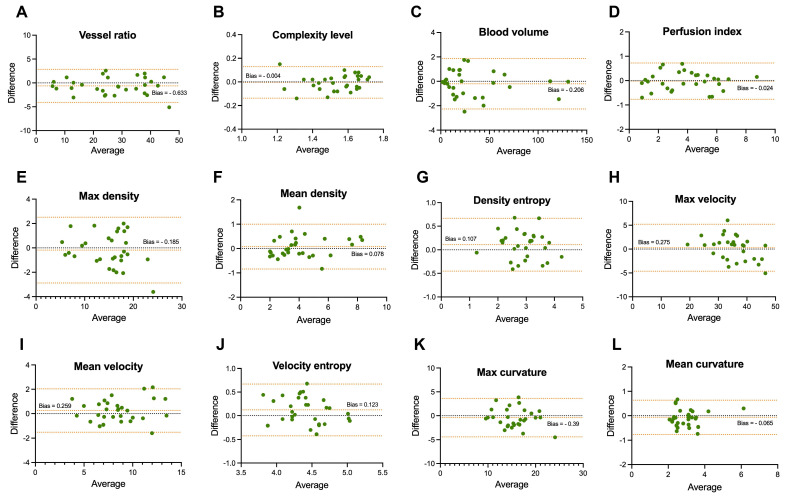
** Inter-observer agreement of ULM structural-functional parameters assessed by Bland-Altman analysis.** (A-D) Hemodynamic and vascular morphological parameters; (E-G) density parameters; (H-J) velocity parameters; (K-L) curvature parameters. Each data point represents a single lesion (n = 30). The central dotted line indicates the mean inter-reader difference (bias), and the upper and lower dashed lines denote the 95% limits of agreement. ULM, ultrasound localization microscopy.

Following manual ROI delineation on grayscale US and mapping of the ROI contours onto the ULM images, all ULM parameters showed high inter-observer agreement. The ICCs ranged from 0.876 to 0.988, indicating excellent reproducibility between the two operators. Bland-Altman analysis revealed no substantial systematic bias. The mean differences for all parameters were close to zero. The majority of measurements fell within the 95% limits of agreement (**Figure 5**).

### Establishment of a reproducible ULM framework for HCC

**Figure 6:**
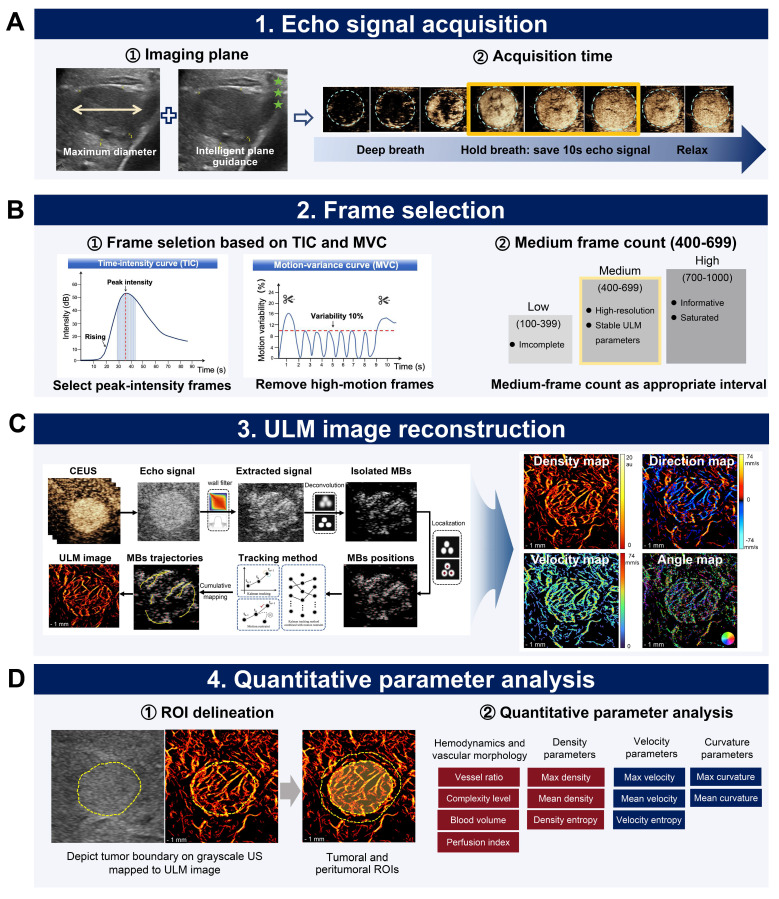
** Reproducible ULM framework for HCC microvascular assessment.** (A) Echo signal acquisition: The imaging plane is fixed by maximum tumor diameter on grayscale US and intelligent plane guidance. A 10-second echo-signal is acquired during a breath-hold initiated at near-complete microbubble filling; (B) Frame selection: Frames are first restricted to the peak-intensity phase identified by the TIC, then screened by MVC to exclude those with motion variability > 10%. The medium-frame count (400–699 frames) is adopted as the reconstruction interval; (C) ULM image reconstruction: Individual MBs are detected and their trajectories are tracked across consecutive frames. ULM images are reconstructed based on the localized MB positions and motion paths. Density, direction, velocity and angle maps are generated. (D) Quantitative parameter analysis: ROIs are delineated on grayscale US to define intratumoral and peritumoral regions. ULM quantitative parameters are extracted from ROIs to objectively assess tumor vasculature. CEUS, contrast-enhanced ultrasound; ULM, ultrasound localization microscopy; HCC, hepatocellular carcinoma; TIC, time-intensity curve; MVC, motion-variance curve; MB, microbubble; ROI, regions of interest. Scale bar: 1 mm.

Based on the preceding analyses, we established a final ULM framework for HCC. The protocol consists of four sequential steps (**Figure 6**). (1) Echo signal acquisition: The optimal imaging plane containing the maximum tumor diameter was identified on grayscale US. This plane was then confirmed by the system’s intelligent plane guidance function (green indicator). During the arterial phase of CEUS, patients took a deep breath upon initial visualization of microbubbles entering the tumor and held their breath once near-complete tumor filling was observed. A 10-second cineloop was acquired during this breath-hold and served as the raw data for ULM reconstruction. Then the patient was asked to resume normal breathing. The CEUS process continued for a total of six minutes (**Figure 6A**); (2) Frame selection: From the 1000-frame acquisition, frames for reconstruction were selected based on two criteria: (i) a temporal window centered on the peak perfusion phase was defined using the TIC, and (ii) within this window, frames with motion variability ≤ 10% as determined by the MVC were retained. Based on the preceding analyses of ULM resolution and parameter repeatability, the medium-frame interval (400–699 frames, corresponding to 4-7 seconds of stable acquisition) was adopted as the optimized reconstruction window (**Figure 6B**); (3) ULM image reconstruction: ULM images (density, velocity, direction, and angle maps) were reconstructed from the selected frames using the built-in toolkit, with all reconstruction performed directly on the clinical ultrasound system and no offline processing required (**Figure 6C**); (4) Quantitative parameter extraction: Intratumoral and peritumoral (0.5 cm annular extension) ROIs were delineated on grayscale US and mapped to the ULM image. ULM parameters were computed separately for each compartment, encompassing four categories: hemodynamic and morphological parameters (vessel ratio, complexity level, blood volume, perfusion index); density parameters (maximum density, mean density, density entropy); velocity parameters (maximum velocity, mean velocity, velocity entropy); and curvature parameters (maximum curvature, mean curvature) (**Figure 6D**).

### Correlation between CEUS metrics and ULM parameters

**Figure 7:**
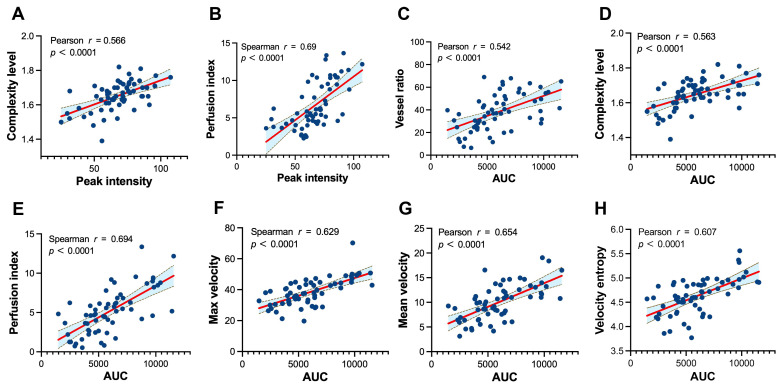
** Correlation between CEUS metrics and ULM parameters.** (A, B) Relationship between CEUS peak intensity and ULM morphological parameters (complexity level, perfusion index); (C-H) Relationship between CEUS AUC and ULM hemodynamic and velocity parameters. Pearson or Spearman correlation coefficients (*r*) and corresponding *p* values are displayed within each panel (n = 61). ULM, ultrasound localization microscopy; CEUS, contrast-enhanced ultrasound; AUC, area under the curve.

Based on the ULM framework described above, we performed correlation analyses between the derived ULM parameters and CEUS quantitative metrics. CEUS peak intensity correlated with both ULM complexity level and perfusion index (**Figure 7A, B**). CEUS AUC demonstrated moderate-to-strong correlations with ULM hemodynamic parameters, including vessel ratio, complexity level and perfusion index. It also correlated with velocity parameters, consisting of maximum velocity, mean velocity and velocity entropy. These correlations are shown in **Figure 7C-H**. These findings confirm that ULM parameters obtained by the established protocol reflect quantifiable perfusion-related characteristics of tumor microvasculature.

### ULM parameters for predicting differentiation grade and MVI in HCC

The demographic and clinical characteristics of the 61 enrolled patients are summarized in **Table 1**. The cohort included 11 patients with p-HCC and 50 with w-HCC. Regarding MVI, 30 patients were MVI–positive and 31 were MVI-negative. HBeAg positivity was more frequent in p-HCC than in w-HCC (45.5% vs. 6.0%, *p* = 0.003). MVI-positive tumors exhibited larger diameters [5.0 cm (3.5-6.6) vs. 3.6 cm (2.9-5.2), *p* = 0.034], lower rates of HBeAb positivity (40.0% vs. 67.7%, *p* = 0.030), and a higher proportion of AFP ≥ 20 ng / mL (66.7% vs. 32.3%, *p* = 0.007).

**Figure 8:**
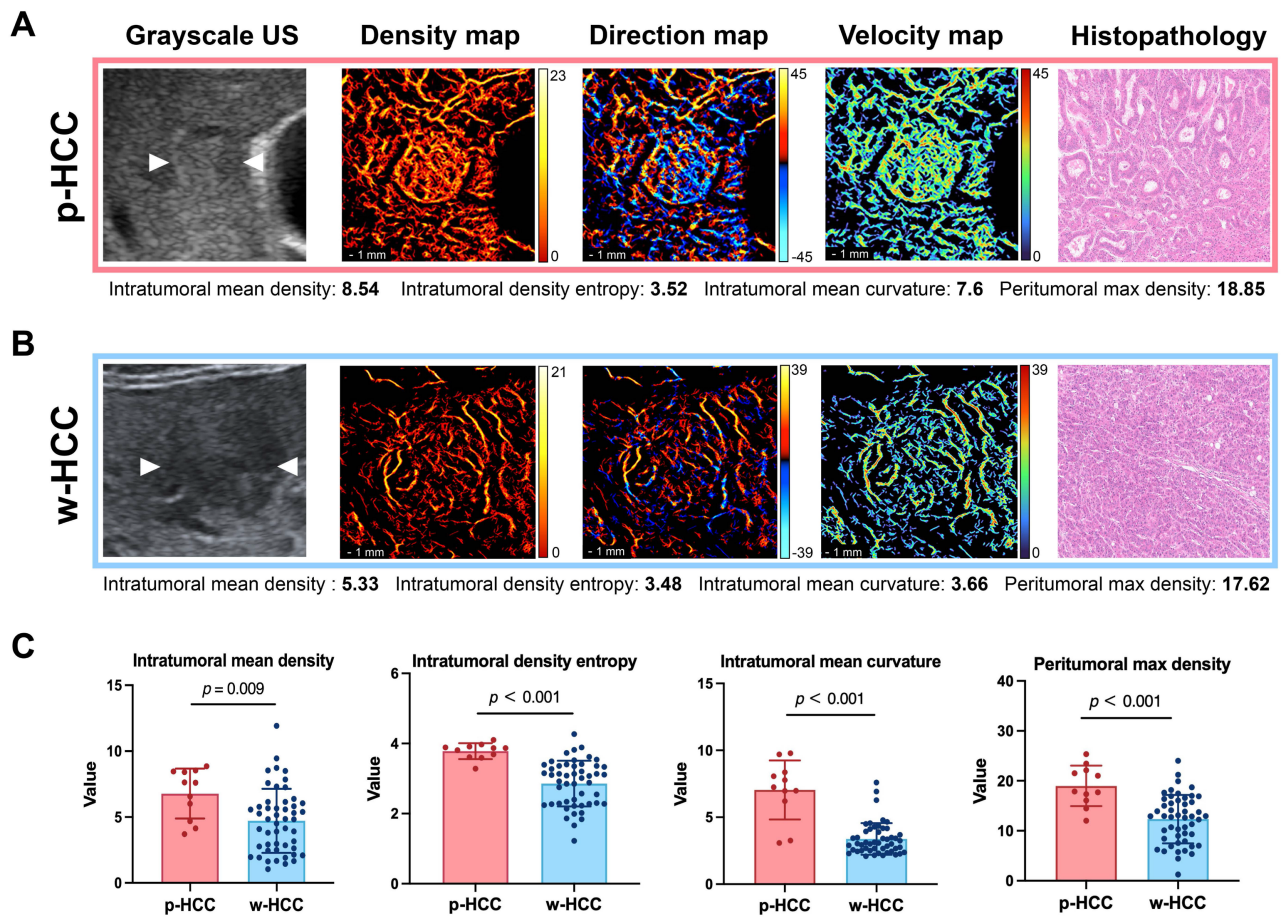
** Comparison of ULM parameters between p-HCC and w-HCC.** (A) Representative case of p-HCC in a 61-year-old male. Grayscale US shows a hypoechoic lesion in segment VI (arrows). ULM maps were reconstructed from 620 frames. Photomicrograph (H&E stain, ×200) confirms p-HCC (ES grade III). (B) Representative case of w-HCC in a 57-year-old male. Grayscale US shows a hypoechoic lesion in segment VIII (arrows). ULM maps were generated from 650 frames. Photomicrograph (H&E stain, ×200) shows w-HCC (ES grade II). (C) Quantitative comparisons of intratumoral mean density, density entropy, mean curvature, and peritumoral maximum density between p-HCC (n = 11) and w-HCC (n = 50). p-HCC, poorly differentiated hepatocellular carcinoma; w-HCC, well-differentiated hepatocellular carcinoma; ULM, ultrasound localization microscopy; H&E, hematoxylin-eosin. Scale bar: 1 mm.

**Figure 9:**
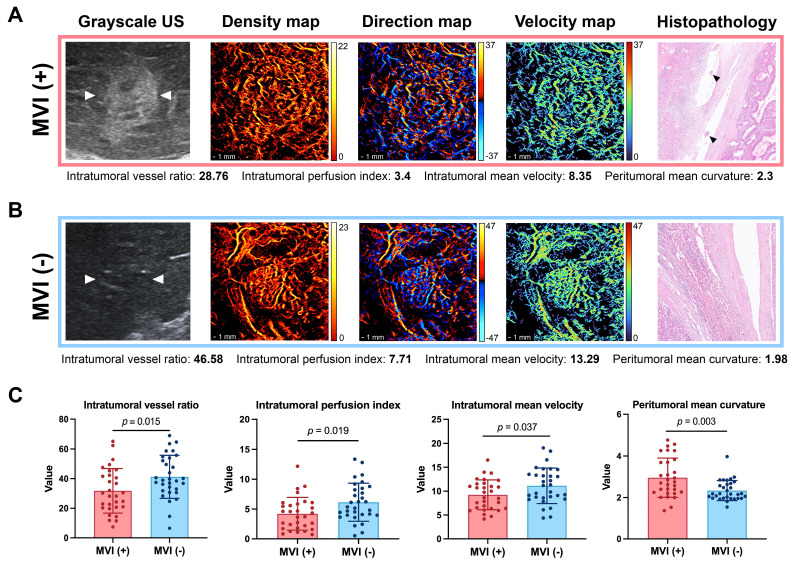
** Comparison of ULM parameters between MVI-positive and MVI-negative HCC.** (A) Representative case of MVI-positive HCC in a 53-year-old male. Grayscale US shows a hypoechoic lesion in segment VI (white arrows). ULM maps were reconstructed from 630 frames. Photomicrograph (H&E stain, ×100) confirms MVI (black arrows). (B) Representative case of MVI-negative HCC in a 50-year-old male. Grayscale US shows a hypoechoic lesion in segment VII (white arrows). ULM maps were reconstructed from 625 frames. Photomicrograph (H&E stain, ×100) shows no evidence of MVI. (C) Quantitative comparisons of intratumoral vessel ratio, perfusion index, mean velocity, and peritumoral mean curvature between MVI-positive (n = 30) and MVI-negative (n = 31) HCC. MVI, microvascular invasion; ULM, ultrasound localization microscopy; H&E, hematoxylin-eosin. Scale bar: 1 mm.

CEUS quantitative parameters did not differ significantly by differentiation grade or MVI status. In contrast, ULM parameters revealed distinct intratumoral and peritumoral alterations (**Note S7**). P-HCCs exhibited significantly higher intratumoral mean density (*p =* 0.009), density entropy (*p* < 0.001), mean curvature (*p* < 0.001), and peritumoral maximum density (*p* < 0.001) (**Figure 8C**). MVI-positive HCCs displayed reduced intratumoral vessel ratio (*p =* 0.015), perfusion index (*p =* 0.019), and mean velocity (*p =* 0.037), but elevated peritumoral mean curvature (*p =* 0.003) (**Figure 9C**). Representative cases are shown in **Figure 8A-B** and **Figure 9A-B**.

ULM parameters with a *p*-value of less than 0.05 were subsequently entered into the univariate and multivariate analyses. Higher intratumoral mean curvature was independently associated with p-HCC (OR 3.36, 95% CI: 1.14-9.85) (**Table 2**). Higher peritumoral mean curvature independently predicted MVI status (OR 12.14, 95% CI: 3.12-47.23) (**Table 3**). The diagnostic performance of these independent predictors is presented in **Table 4**. Intratumoral mean curvature to predict p-HCC yielded a sensitivity of 81.8%, specificity of 94.0%, accuracy of 91.8%, and an AUC of 0.91 (95% CI: 0.80-0.99). To address the imbalanced sample sizes of the differentiation groups, a 1:2 random subsampling analysis was performed and is reported in **Note S8**. For MVI prediction, the peritumoral mean curvature showed a sensitivity of 90.0%, specificity of 58.1%, accuracy of 73.8%, and an AUC of 0.78 (95% CI: 0.66-0.90).

## Discussion

Preoperative assessment of HCC differentiation and MVI remains a significant clinical challenge. ULM has made it possible to depict the microcirculation of intratumoral and peritumoral regions. However, the application of ULM is limited by technical variability, which arises from the absence of a uniform imaging workflow 25, 26, 33. This study established a reproducible framework for ULM imaging and analysis, enabling high-quality visualization of microvascularity and ensuring the repeatability of ULM quantitative parameters.

In recent years, ULM has rapidly entered clinical diagnostics and is now being applied across a variety of organs and diseases to extract quantitative microvascular parameters for clinical decision-making. Li et al. performed testicular ULM and successfully differentiated non-obstructive from obstructive azoospermia using ULM-derived metrics 34. Bodard et al. used sensing ULM to characterize renal pseudotumors, showing that it can distinguish benign from malignant lesions 35. Denis et al. applied transcranial ULM in moyamoya disease, revealing deep cerebral microvascular abnormalities not visible with conventional imaging 36. Liu et al. used a clinically practical protocol, with 100 Hz frame rate and 15-second acquisition time, to resolve tibial nerve microcirculation down to 10 μm. This approach also detected significant differences in diabetic patients 37. These studies confirm that ULM can provide clinically meaningful microvascular biomarkers for various diseases. Notably, recent studies have employed clinical ultrasound scanners and post-processing frameworks. This shift shows that ULM is no longer limited to offline analysis.

However, the heterogeneity in acquisition protocols and analysis methods greatly impact the comparability of ULM parameters. This poses a critical barrier to widespread clinical use. In this study, we establish a reproducible ULM framework for HCC and provide a reference workflow for future multi-center studies. Previous ULM studies conventionally acquire data during the late wash-out phase to ensure microbubble sparsity 18. For HCC, however, we found that peak-intensity acquisition revealed a more complete tumor microvascular network than wash-out phase imaging (**Note S9**). HCC is predominantly supplied by the hepatic artery and enhances rapidly during the arterial phase. Peak-intensity phase reflects the most complete tumor perfusion. During the wash-out phase, most microbubbles clear rapidly, and the residual sparse bubbles primarily originate from slow-flow vessels. Consequently, many tumor-feeding vessels become undetectable, leading to underestimation of vascular volume and reduced diagnostic sensitivity. Therefore, we assume that the HCC ULM image at peak-enhancement phase provides a more faithful representation of the tumor microvasculature.

The appropriate frame counts for ULM reconstruction also need careful consideration. Given a median HCC depth of 5.1 cm (IQR, 4.2-6.2) in our study, a convex-array probe (S1-8C) with a frame rate of 100-150 Hz was necessary to preserve acceptable image quality. Moreover, most patients could sustain a breath-hold for less than 10 seconds. Under these clinical constraints of deep abdominal imaging, we consider that a 1000-frame acquisition (100 Hz, 10 seconds) represents a practical protocol that balances ULM quality, patient compliance, and data reliability (**Note S10**). As the frame count increased, we found that the vessel counts growth rate slowed after approximately 700 frames. Meanwhile, the minimum vessel diameter and inter-vessel distance reached their lowest values at around 600 frames, and then gradually increased. We assume that those trends can be attributed to two factors. Firstly, the accumulation of numerous localizations near vessel walls produces a blooming-like diffusion effect. Even with a diluted microbubble method, inherent localization uncertainty persists. Moreover, when the inter-bubble distance falls below 1.7–3 times the point spread function, interference can cause localization errors 38. It progressively overestimates vessel diameter. Secondly, despite breath-holding, micro-displacements induced by cardiac pulsation or gradual diaphragmatic relaxation still occur. Residual motion accumulation during breath-holding inevitably introduces slight misregistration, blurring the smallest inter-vessel gaps 39. Regarding the structural–functional parameters, the hemodynamic and density parameters initially increased and then plateaued with frame count. Velocity and curvature parameters remained stable across frame counts. These patterns can be explained as follows. Hemodynamic and density parameters essentially reflect the whole spatial coverage of the vascular network. In the low-frame regime, insufficient localization accumulation leads to underestimation of density and perfusion metrics. When the frame count reaches the medium range, the main vascular network is largely reconstructed, and these parameters approach saturation. Further accumulation in the high-frame range primarily fills sparsely perfused terminal branches or re-samples already filled vessels, contributing marginally to the overall perfusion indices. Velocity parameters are derived from tracking individual microbubbles over consecutive frames and fitting their local trajectories. As long as the trajectory continuity is preserved, velocity measurements remain unaffected by changes in localization density. Curvature parameters describe the local bending of the vessel centerline and depend solely on the geometric trajectory of the vessel itself, independent of the number of localizations. Hence, curvature remains stable across frame groups. Taken together, in the low-frame counts, localization accumulation is insufficient, resulting in lower vessel counts, and reduced density and perfusion parameters 40. In the medium-frame regime, the main vascular network is largely filled, with both the high spatial resolution and stable structural–functional parameters. In the high-frame counts, minimum vessel diameter and inter-vessel distance slightly rebound, whereas structural–functional parameters remain stable. Velocity and curvature are essentially frame-count-independent. Considering the practical constraint of breath-hold duration in clinical practice, we conclude that 400-699 frames (4-7 seconds at 100 Hz) represents an appropriate frame-count interval that balances spatial resolution with reproducible structural-functional parameters.

Although ULM-derived parameters have been applied in several clinical studies, their consistency and repeatability remain insufficiently verified 41, 42. In this study, we confirm that ULM parameters demonstrate excellent agreement, which is consistent with prior reports 43. While CEUS time-intensity curves also provide perfusion-related parameters 44, 45, our analysis reveals moderate-to-strong correlations between certain ULM parameters and CEUS metrics related to perfusion and velocity. This suggests that ULM and CEUS capture related yet distinct aspects of tumor hemodynamics.

Notably, while CEUS metrics showed no significant association with pathological grade or MVI status, several ULM parameters demonstrated distinct differences, suggesting that the superior spatial resolution of ULM enables more detailed quantification of tumor microvascular architecture. For discriminating p-HCC, intratumoral mean curvature yielded a sensitivity of 81.8%, a specificity of 94.0%, an accuracy of 91.8%, and an AUC of 0.91. This performance exceeds that of contrast-enhanced CT parameters such as the portal relative enhancement ratio (PRER), which achieved an AUC of only 0.574 46. By comparison, the ADC_slow derived from intravoxel incoherent motion diffusion-weighted MRI, a metric reflecting cellular density and extracellular space restriction, reached a comparable diagnostic accuracy (sensitivity 91.4%, specificity 91.1%) 47. Biologically, poor differentiation is characterized by increased cell density, nuclear pleomorphism, and architectural disorganization. These changes not only restrict water diffusion and diminish portal venous enhancement due to sinusoidal loss and distortion, but also profoundly alter the tumor microvasculature. Rapidly proliferating p-HCC cells give rise to markedly tortuous and irregularly branched neovessels 45. Immature tumor vasculature often appears as simplified linear or dilated sinusoidal channels, a pattern that directly manifests as higher vascular density and elevated curvature on ULM. Thus, intratumoral mean curvature serves as a quantitative imaging biomarker reflecting the microvascular disorganization inherent to poor differentiation.

Regarding MVI, peritumoral mean curvature achieved a sensitivity of 90.0%, a specificity of 58.1%, an accuracy of 73.8%, and an AUC of 0.78. This performance is higher than that of CT-derived arterial absolute enhancement value (AUC 0.557) and PRER (AUC 0.572) 46. A combined PET/CT and serum AFP model yielded a comparable AUC of 0.726 but with lower sensitivity (56.9%) and higher specificity (83.9%) 48. Among MRI-based approaches, the VICT2 trait combining peritumoral hypoenhancement, incomplete capsule, corona enhancement, and T2 hyperintensity demonstrated an accuracy of 73%, similar to our method 49. MR elastography assessing tumor-to-liver shear wave speed ratio achieved AUCs of 0.84-0.86 50, which are higher than that of ULM. MVI is defined by tumor cell invasion into peritumoral microvessels, a process that promotes excessive immature neovascularization, producing markedly tortuous and disorganized microvessels 51. Elevated peritumoral curvature therefore quantitatively captures the microvascular tortuosity driven by invasive growth, supporting its potential role as a novel imaging biomarker for MVI assessment.

Collectively, these findings demonstrate that ULM parameters derived by a reproducible framework can serve as non-invasive predictors of pathological grade and MVI in HCC. Our study still has several limitations. First, the single-center design, modest sample size, and the resultant imbalance in HCC subgroups may constrain the generalizability of the findings. External validation through multicenter studies is warranted. Second, ULM acquisition was performed only during the CEUS peak-intensity phase. Extending imaging to multiple phases might capture time-dependent vascular dynamics and improve diagnostic accuracy. Third, although intra-method and inter-observer reproducibility were assessed, variability across imaging sessions, operators, or US systems was not evaluated. Investigating these factors will be essential for ensuring robustness in broader clinical implementation.

## Conclusions

This study establishes a reproducible ULM framework for HCC that demonstrates high repeatability and excellent inter-observer agreement. The ULM-derived quantitative parameters provide a comprehensive assessment of tumor microvascularity and show promise as non-invasive biomarkers for the preoperative evaluation of tumor differentiation and MVI status.

## Supplementary Material

Supplementary methods, figures and tables.

## Figures and Tables

**Table 1:** Characteristics of HCC participants

Characteristics	Differentiation grade				MVI status			
	p-HCC (n = 11)	w-HCC (n = 50)	*p* value		Positive (n = 30)	Negative (n = 31)	*p* value	
**Age (years)**	57.18 ± 7.31	58.46 ± 8.51	0.646		57.60 ± 8.56	58.84 ± 8.06	0.563	
**Gender**			1.000				1.000	
Male	10 (90.9%)	43 (86%)			26 (86.7%)	27 (87.1%)		
Female	1 (9.1%)	7 (14%)			4 (13.3%)	4 (12.9%)		
**Tumor size (cm)**	4.91 (4.20, 6.61)	3.95 (3.13, 5.65)	0.136		5.00 (3.51, 6.60)	3.62 (2.90, 5.23)	**0.034**	
**Cirrosis**	5 (45.5%)	12 (24%)	0.287		10 (33.3%)	7 (22.6%)	0.349	
**HBsAg positive**	10 (90.9%)	33 (66%)	0.202		20 (66.7%)	23 (74.2%)	0.519	
**HBsAb positive**	1 (9.1%)	9 (18%)	0.785		26 (86.7%)	25 (80.6%)	0.772	
**HBeAg positive**	5 (45.5%)	3 (6%)	**0.003**		6 (20%)	2 (6.5%)	0.235	
**HBeAb positive**	5 (45.5%)	28 (56%)	0.525		12 (40%)	21 (67.7%)	**0.030**	
**HBcAb positive**	10 (90.9%)	43 (86%)	1.000		24 (80%)	29 (93.5%)	0.235	
**Laboratory values**								
AFP ≥ 20 ng/mL	7 (63.6%)	23 (46%)	0.289		20 (66.7%)	10 (32.3%)	**0.007**	
NLR	2.33 ± 1.14	2.33 ± 1.03	0.985		2.38 ± 0.91	2.28 ± 1.17	0.707	
PLR	100.00 (74.59, 145.74)	96.71 (81.26, 118.61)	0.694		98.65 (89.42, 133.45)	90.64 (68.51, 111.59)	0.108	

Note: HCC, hepatocellular carcinoma; MVI, microvascular invasion; p-HCC, poorly-differentiated HCC; w-HCC, well-differentiated HCC; AFP, α-fetoprotein; HBsAg, Hepatitis B surface antigen; HBsAb, Hepatitis B surface antibody; HBeAg, Hepatitis B e antigen; HBeAb, Hepatitis B e antibody; HBcAb, Hepatitis B core antibody; NLR = neutrophil count (× 10^9^ / L) / lymphocyte count (× 10^9^ / L). PLR = platelet count (× 10^9^ / L) / lymphocyte count (× 10^9^ / L)

**Table 2:** Univariate and multivariate logistic regression analyses of ULM parameters for predicting poorly-differentiated HCC.

Parameters	Univariate analysis			Multivariate analysis	
	Odds ratio (95% CI)	*p* value		Odds ratio (95% CI)	*p* value
Intratumoral mean density	1.09 (0.99 - 1.20)	0.094		-	-
Intratumoral density entropy	1.44 (1.06 - 1.95)	**0.018**		0.63 (0.12 - 3.32)	0.582
Intratumoral mean curvature	2.74 (1.65 - 4.54)	**< 0.001**		3.36 (1.14 - 9.85)	**0.027**
Peritumoral max density	1.40 (1.14 - 1.72)	**0.002**		1.56 (0.90 - 2.69)	0.113

Note: ULM, ultrasound localization microscopy; HCC, hepatocellular carcinoma; CI, confidential interval.

**Table 3:** Univariate and multivariate logistic regression analyses of ULM parameters for predicting MVI in HCC.

Parameters	Univariate analysis			Multivariate analysis	
	Odds ratio (95% CI)	*p* value		Odds ratio (95% CI)	*p* value
Intratumoral vessel ratio	0.96 (0.92 - 0.99)	**0.021**		1.01 (0.85 - 1.20)	0.928
Intratumoral perfusion index	0.80 (0.66 - 0.97)	**0.020**		0.55 (0.12 - 2.47)	0.435
Intratumoral mean velocity	0.85 (0.73 - 0.99)	**0.042**		1.27 (0.62 - 2.61)	0.511
Peritumoral mean curvature	5.01 (1.80 - 13.97)	**0.002**		12.14 (3.12 - 47.23)	**< 0.001**

Note: ULM, ultrasound localization microscopy; MVI, microvascular invasion; HCC, hepatocellular carcinoma; CI, confidential interval.

**Table 4:** Diagnostic performance of ULM parameters for predicting differentiation grade and MVI status in HCC

Parameters	Sensitivity	Specificity	Accuracy	AUC (95% CI)
Intratumoral mean curvature to diagnose p-HCC	81.8%	94.0%	91.8%	0.91 (0.80 - 0.99)
Peritumoral mean curvature to diagnose MVI status	90.0%	58.1%	73.8%	0.78 (0.66 - 0.90)

Note: ULM, ultrasound localization microscopy; MVI, microvascular invasion; p-HCC, poorly-differentiated hepatocellular carcinoma; CI, confidential interval.

## Data Availability

Data supporting the findings of this study may be obtained from the corresponding author upon reasonable request.

## Abbreviations

AFP: α-fetoprotein
CEUS: contrast-enhanced ultrasound
CI: confidential interval
COV: coefficient of variation
HCC: hepatocellular carcinoma
H&E: hematoxylin-eosin
MB: microbubble
MVC: motion-variance curve
MVI: microvascular invasion
p-HCC: poorly-differentiated hepatocellular carcinoma
ROI: region of interest
TIC: time-intensity curve
ULM: ultrasound localization microscopy
US: ultrasound
w-HCC: well-differentiated hepatocellular carcinoma
