# Supplementary material for: A reproducible ultrasound localization microscopy framework for the quantitative imaging of hepatocellular carcinoma on a clinical ultrasound system
Source: Theranostics. 2026 Jul 20;16(14):8230–46. doi: 10.7150/thno.133561 (PMC13440603; doi:10.7150/thno.133561)
Supplement: Supplementary file 1 [file thnov16p8230s1.pdf]

Supplementary material

Note S1. Intelligent imaging plane guidance

To address motion caused by unintentional operator movement or tissue deformation due to applied probe pressure, the system integrates a plane guidance method. During data acquisition, it intelligently extracts texture and edge features from the current scanning image and matches them with the corresponding features of a predefined ideal scanning plane. The degree of matching is displayed as a color-coded indicator near the scanning image (red = no match, green = match) (**Figure S1**). This provides a real-time indication of how well the current scanning plane matches the ideal plane throughout the acquisition process, thereby ensuring the validity of the acquired raw data for ultrasound localization microscopy (ULM) reconstruction.

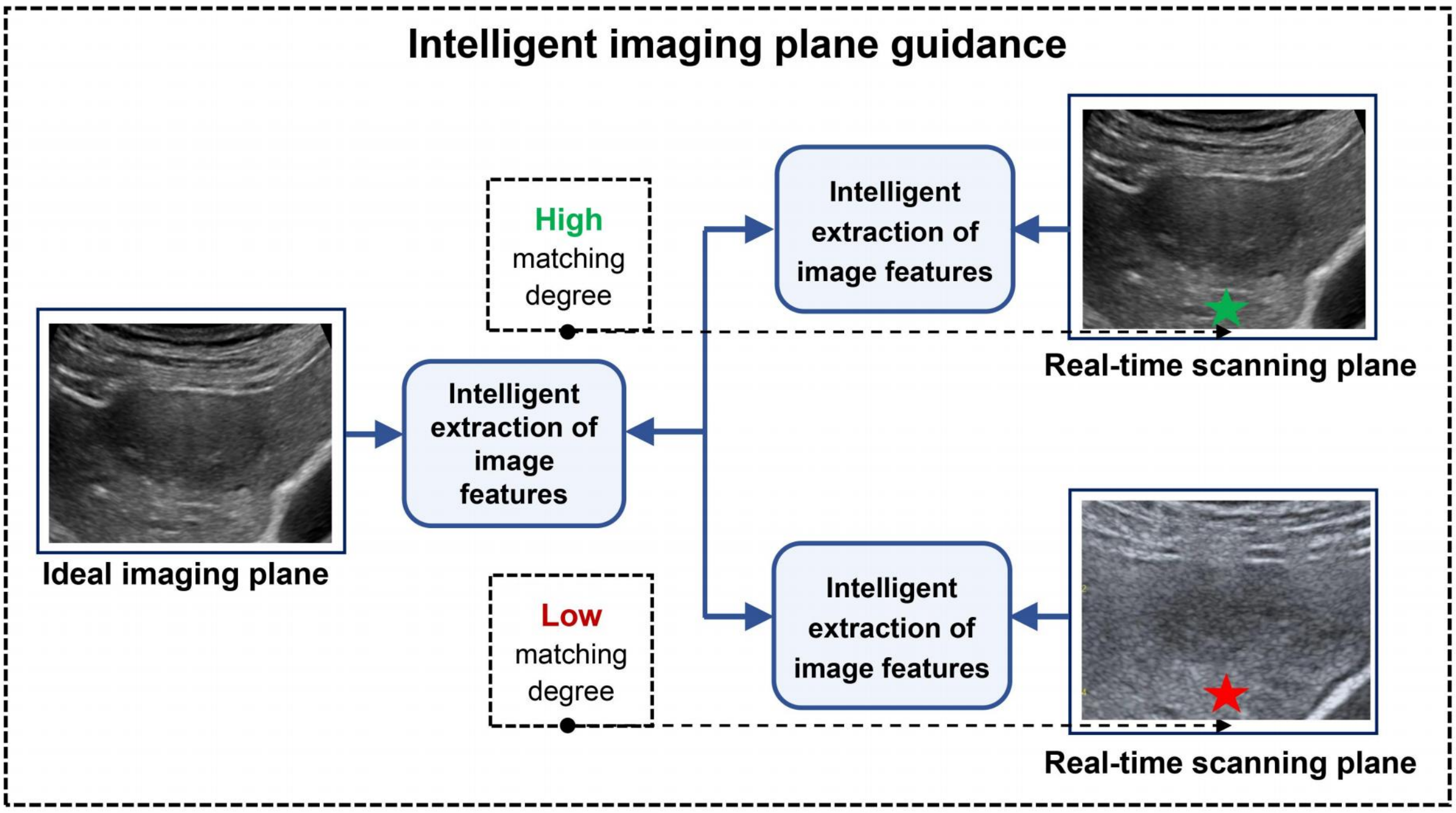

Figure S1. Intelligent imaging plane guidance

Note S2. Calculation method of motion-variance curve

The motion-variance curve (MVC) is a built-in tool of the VINNO ultrasound system (no external device required). After echo signal acquisition is completed, the MVC is automatically generated, providing a visualized curve to guide frame selection. MVC is derived by calculating the frame-to-frame correlation of sequential two-dimensional images

acquired simultaneously with the contrast-enhanced ultrasound (CEUS) data. When the scan plane undergoes rapid motion within a short period, consecutive frames differ substantially, resulting in low correlation and a high peak on the curve. Conversely, when the scan plane remains relatively stable, consecutive frames are similar, yielding high correlation and a low amplitude on the curve.

For two images A and B of the same size (both $M \times N$), their correlation coefficient r is defined as:

$$r = \frac{\sum_{i=1}^{M}\sum_{j=1}^{N}\left(A_{ij} - \bar{A}\right)\left(B_{ij} - \bar{B}\right)}{\sqrt{\left[\sum_{i=1}^{M}\sum_{j=1}^{N}\left(A_{ij} - \bar{A}\right)^2\right]\left[\sum_{i=1}^{M}\sum_{j=1}^{N}\left(B_{ij} - \bar{B}\right)^2\right]}}$$

A_{ij} and B_{ij} represent the pixel values at position (i, j) in images A and B, respectively.
$\bar{A}$ and $\bar{B}$ are the mean pixel values of all pixels in the two images.

$$\bar{A} = \frac{1}{MN}\sum_{i=1}^{M}\sum_{j=1}^{N} A_{ij}, \; \bar{B} = \frac{1}{MN}\sum_{i=1}^{M}\sum_{j=1}^{N} B_{ij}$$

The variability (%) in the MVC is defined as $(1 - r) \times 100\%$. For example, if the correlation coefficient between two consecutive frames is $r = 0.9$, the variability is $1 - 0.9 = 0.1$ (10%). Therefore, in this study, only frames with a motion variability ≤ 10% (correlation ≥ 0.9) were retained for ULM reconstruction, ensuring high stability (**Figure S2**).

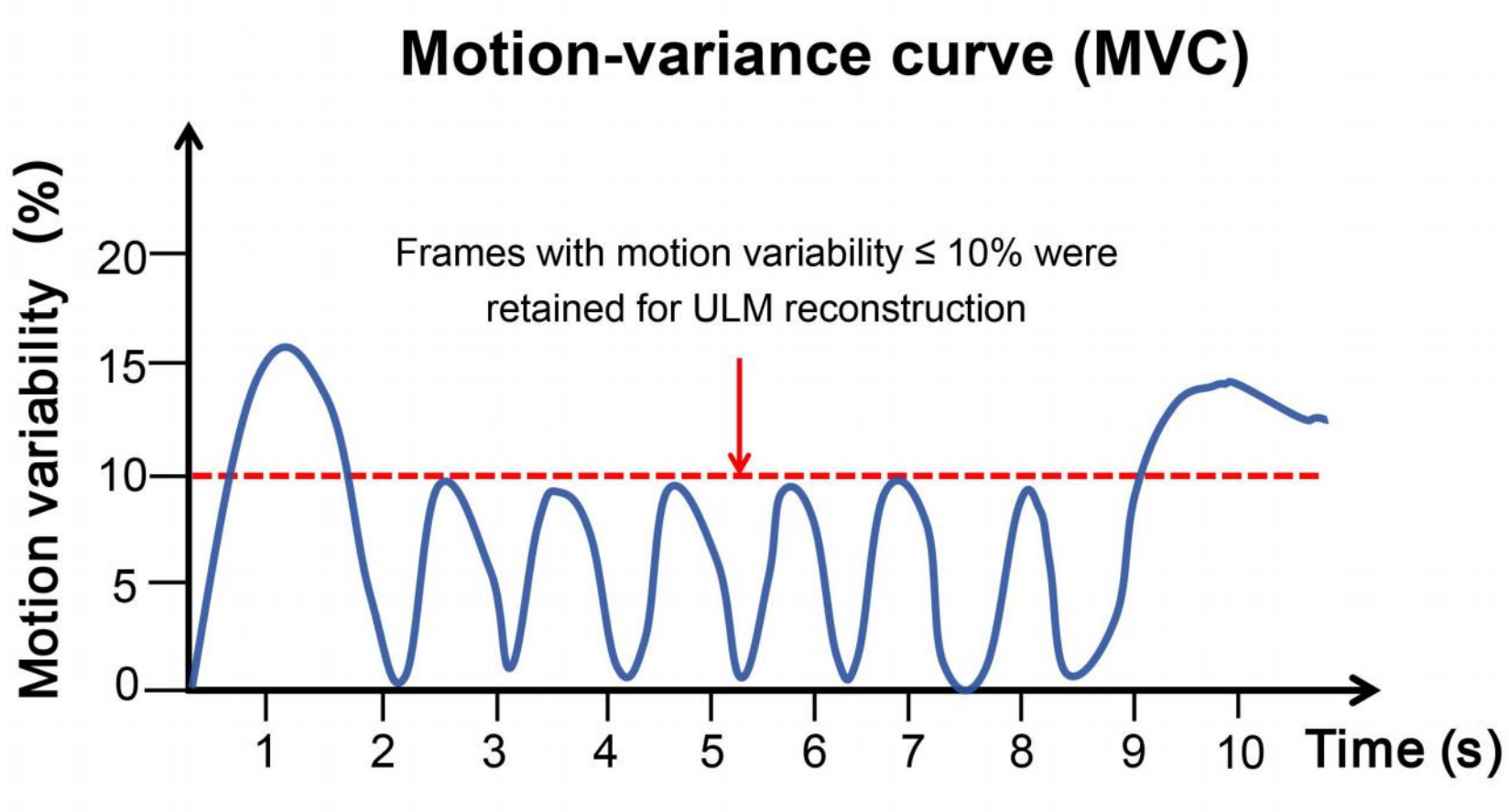

Figure S2. Motion-variance curve (MVC). MVC a built-in tool of the VINNO ultrasound system (no external device required). After echo signal acquisition is completed, the MVC is automatically generated. Only frames with a motion variability ≤ 10% were retained for ULM reconstruction.

Note S3. Calculation of ULM resolution indices and resolution analysis

1. Calculation method of ULM resolution indices

On the vascular density map, a normalized density distribution curve (fitted density curve) is extracted along a line perpendicular to the vessel course. Based on the peak width and inter-peak distance of the curve, quantitative measurements of vessel count, vessel diameter and inter-vessel distance can be achieved (**Figure S3**).

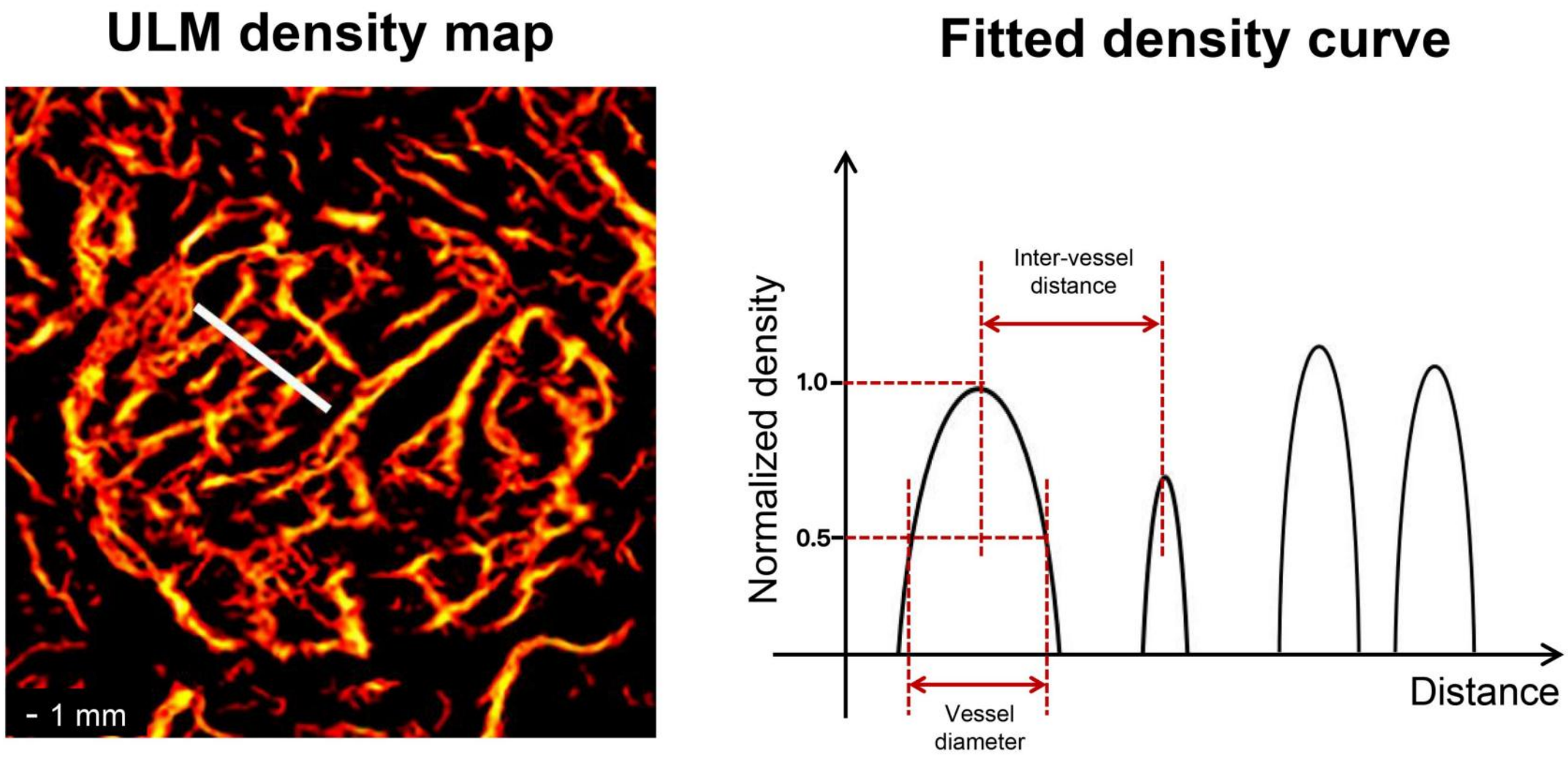

Figure S3. Calculation method for ULM resolution indices. Scale bar: 1 mm.

(1) Vessel count = the number of peaks in the normalized density distribution curve extracted along a line perpendicular to the vessel course on the vascular density map.
(2) Minimum vessel diameter = the smallest full width at half maximum (FWHM) of the vessel cross-sectional density peaks across all measured vessels.
(3) Minimum inter-vessel distance = the smallest distance between adjacent peaks among all vessel pairs in the analyzed region.

2. ULM resolution analysis in this study

The diffraction limit in ultrasound is commonly defined as half the wavelength (λ/2). With a speed of sound in tissue of c ≈ 1540 m/s and the centre frequency of the convex probe was 3.2 MHz, the wavelength is λ = c/f ≈ 481 μm, yielding a diffraction limit of ≈ 240 μm.

To determine whether our ULM images overcome this limit, we performed a ULM resolution analysis. From the 61 HCC cases, we randomly selected 30 cases. We measured the minimum vascular diameter and the minimal inter-vessel distance using fitted density curve (**Figure S4**). **(1) Minimum vessel diameter:** In each tumor, the smallest clearly delineated microvessel was identified on the ULM density map and its diameter was measured; **(2)**

Minimum inter-vessel distance: Each tumor was divided into four quadrants. In each quadrant, well-isolated and unbranched microvessels were chosen from both the centre and the periphery to ensure representative sampling. The minimum inter-vessel distance for a given tumour was the smallest value among four quadrants. Data are reported as mean ± standard deviation if normally distributed, or as median with interquartile range otherwise.

The results showed that the minimum vessel diameter was 91.3 ± 22.7 μm (range: 55-150 μm) and the minimum inter-vessel distance was 168 ± 48.55 μm (range: 100-280 μm), both substantially smaller than the 240 μm diffraction limit. Therefore, these results confirm that the spatial resolution of our ULM images has overcome the diffraction limit, achieving true super-resolution imaging.

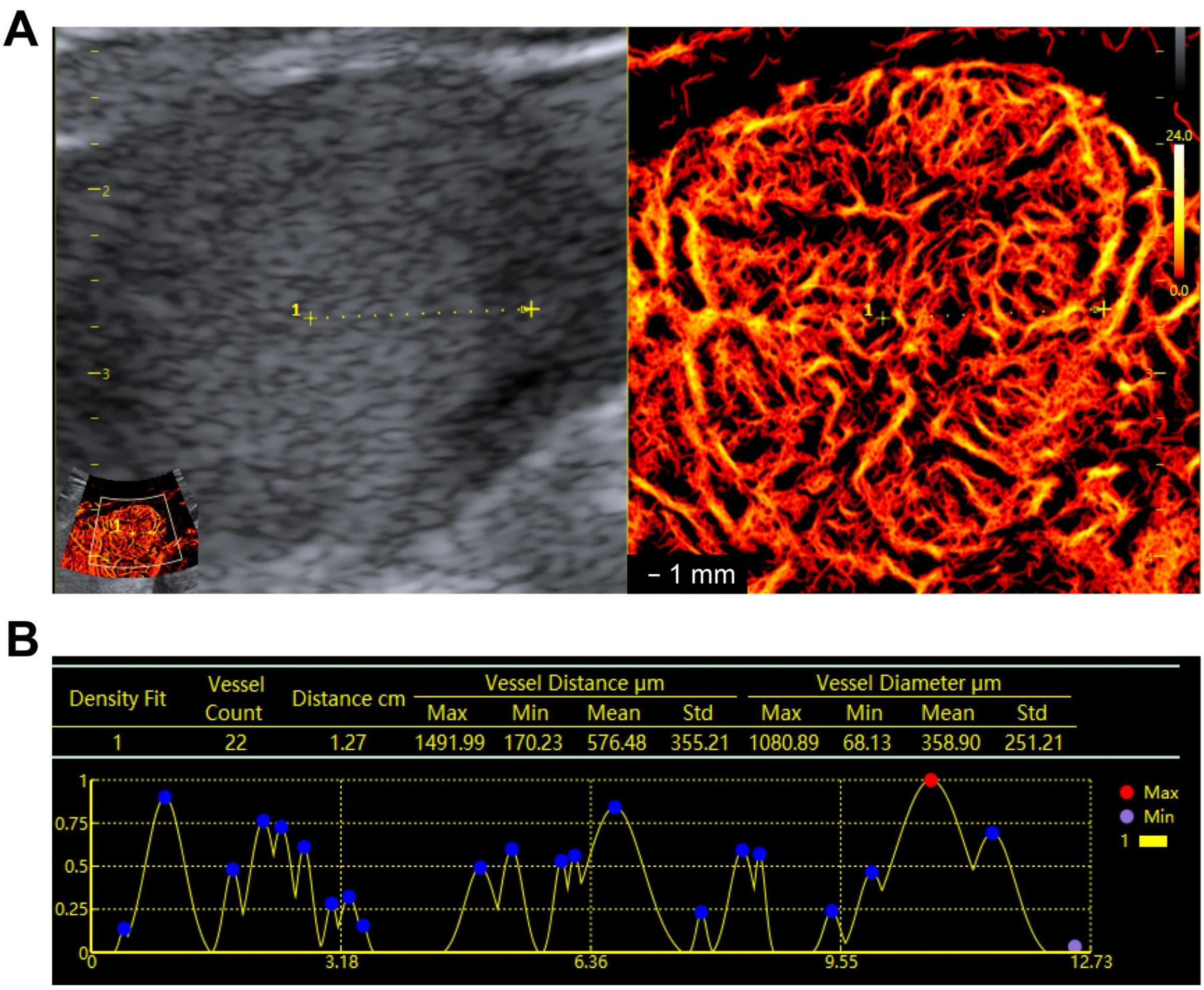

Figure S4. ULM resolution analysis. (**A**) ULM density map of a HCC case. A yellow line is placed to indicate the vascular distribution within a selected tumour region. (**B**) The fitted density curve is generated from the vessel distribution along the yellow line. The minimum vascular diameter is 68.13 μm and the minimum inter-vessel distance is 170.23 μm. Scale bar: 1 mm.

3. Fourier Ring Correlation (FRC) analysis

To overcome the limitations of FWHM, we supplemented our analysis with Fourier Ring Correlation (FRC). As illustrated in **Figure S5**, the procedure is as follows: (1) The list of microbubble trajectories is randomly divided into two independent subsets of roughly equal size, and two sub-images are reconstructed separately to ensure independent spatial frequency content; (2) After 2D Fourier transform of each sub-image, the FRC curve is obtained by computing the normalized correlation along rings of constant spatial frequency. The curve decays from unity (signal-dominated low frequencies) toward zero (noise-dominated high frequencies); (3) Resolution is taken as the reciprocal of the spatial frequency at the intersection of the FRC curve with predefined thresholds. The 2σ threshold curve corresponds to twice the standard deviation of the noise correlation, representing the highest spatial frequency at which the correlation is statistically significantly above the noise level. The 1/2-bit threshold curve indicates the highest spatial frequency at which the information content per pixel drops to 0.5 bit. The FRC curve is 10-point smoothed before intersection detection. If multiple intersections exist, the one corresponding to the lower resolution is selected.

Following the above procedure, resolutions of 48.13 μm (2σ threshold) and 38.04 μm (1/2-bit threshold) were determined. Both values are substantially smaller than the 240 μm diffraction limit (λ/2) of the 3.2 MHz convex array probe, confirming that the resolution of HCC ULM image in this study has overcome the diffraction barrier.

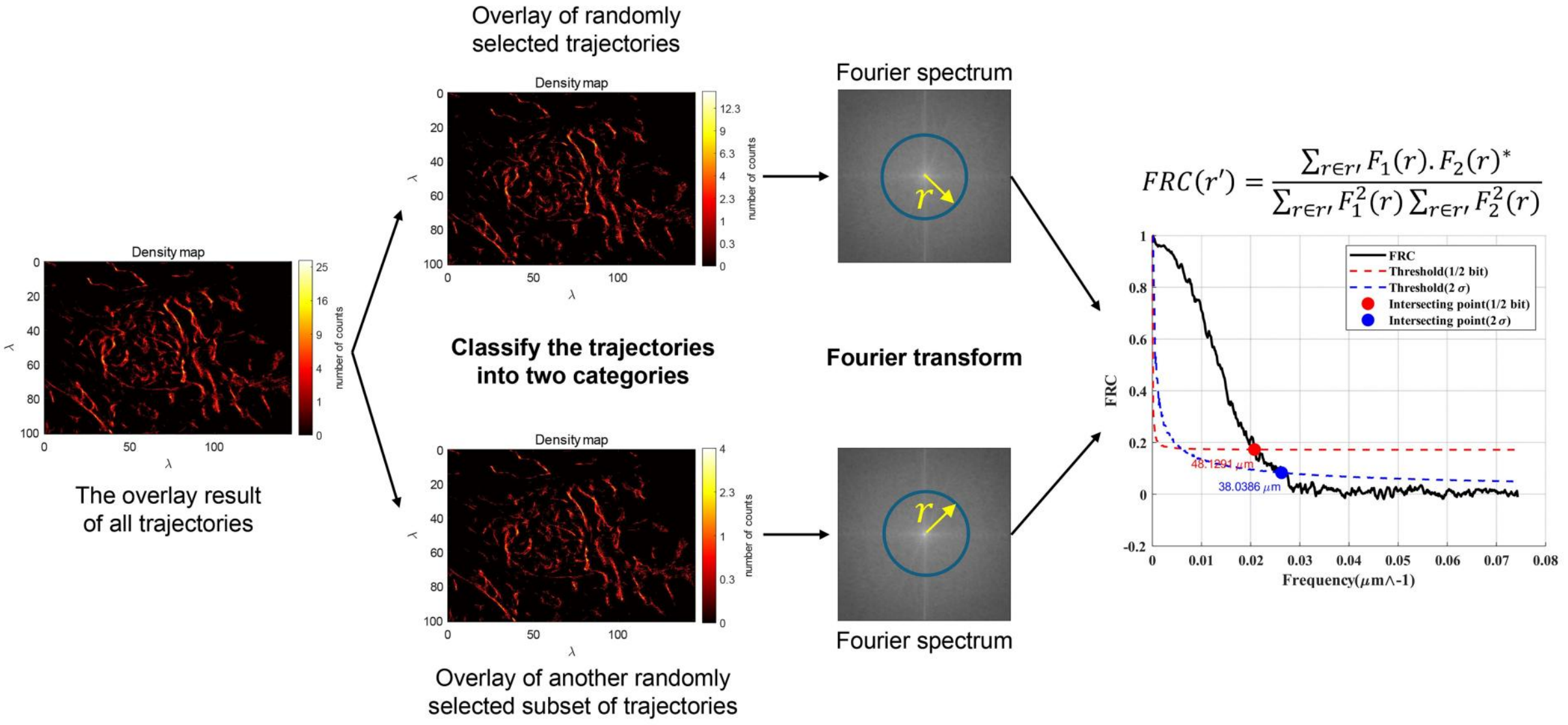

Figure S5. Fourier Ring Correlation (FRC) analysis. The resolution is determined by the intersection of the FRC curve with the 2σ threshold and the 1/2-bit threshold, yielding 48.13 μm and 38.04 μm, respectively.

Note S4. Calculation method of ULM structural-functional parameters

(1) Density parameters (maximum density, minimum density, mean density, Std density)

Density parameters represent the number of microbubbles that have passed through the region of interest (ROI) within a certain period of time. The greater the number of microbubbles passing through that location, the higher the density value at that point. The quantitative parameters related to vascular density are calculated as follows (**Figure S6**):

1) Max density = the maximum vascular density value within the ROI.

2) Min density = the minimum vascular density value among regions with vascular density greater than zero within the ROI.

3) Mean density (MeanDen) = the sum of vascular density values in regions with vascular density greater than zero within the ROI, divided by the number of pixels in those regions.

$$\text{MeanDen} = \frac{\sum_{i \in \{\text{voxels with Den} > 0\}} \text{Den}_i}{n_{\text{Den} > 0}}$$

($n_{\text{Den} > 0}$ is the number of voxels with vascular density greater than zero in the ROI.)

4) Standard deviation of density (Std Den) = the sample standard deviation of vascular density values over pixels with vascular density greater than zero in the ROI.

$$\text{Std Den} = \sqrt{\frac{1}{n-1}\sum_{i=1}^{n}(\text{Den}_i - \text{MeanDen})^2}$$

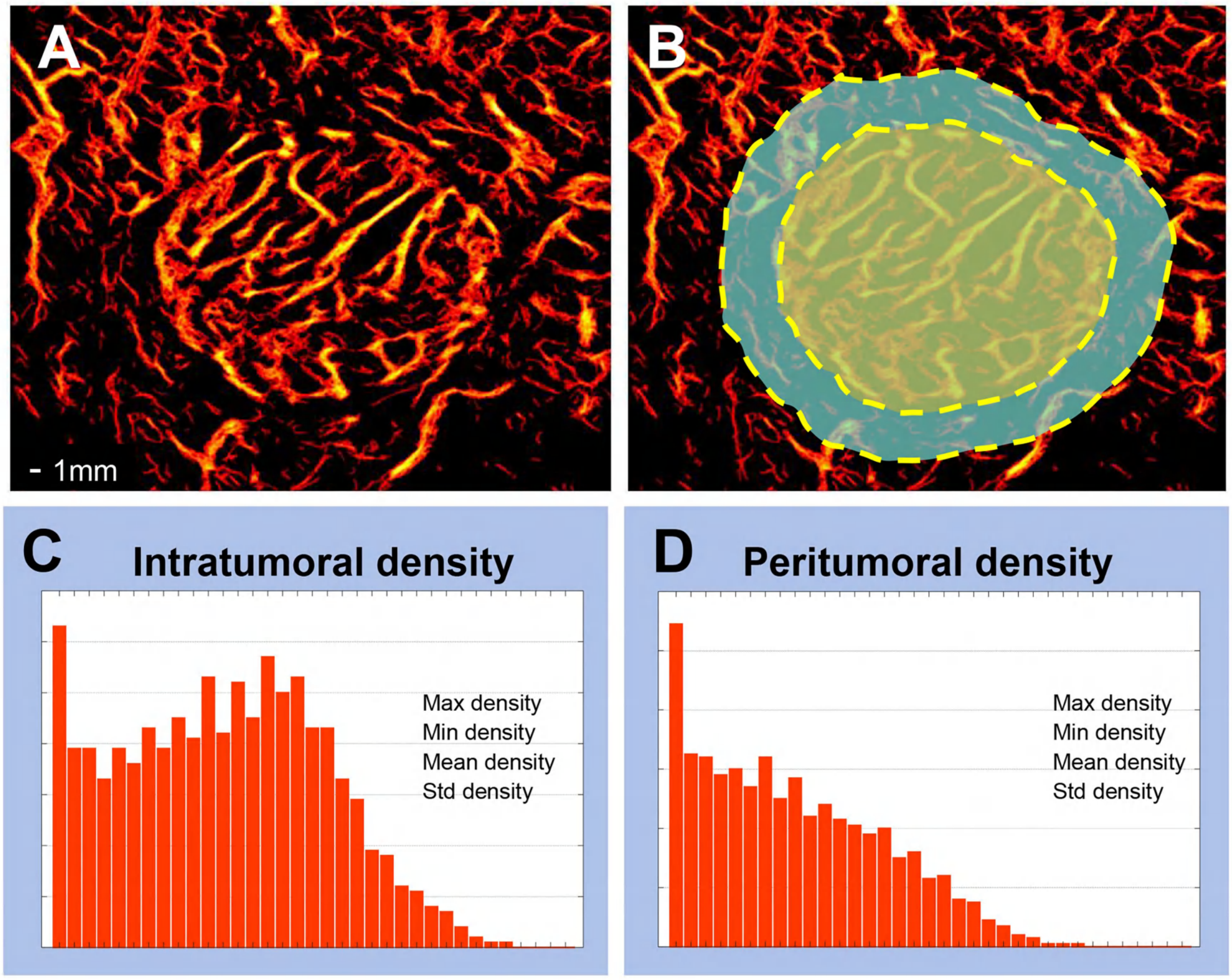

Figure S6. Calculation method for density parameters. (A) Density map; (B) Intratumoral (yellow) and peritumoral 0.5cm (blue) ROIs were depicted; (C-D) Max density, min density, mean density and Std density were calculated by analyzing numerical distribution of density in two regions of ROIs. ROI, region of interest. Std, standard deviation. Scale bar: 1 mm.

(2) Density entropy

Density entropy measures the uniformity of microvessel distribution within the tissue sample. Higher entropy value indicates greater uncertainty or randomness in the system. The ROI is divided into multiple small regions, and the microvascular density in each small region

is measured. The density values are then grouped into several intervals or categories (e.g., low, medium, high density), and the frequency of occurrence for each interval is calculated, which gives the probability p_i (number of samples in the interval divided by the total number of samples) (**Figure S7**).

Entropy is calculated using this formula, where X is a random variable with N possible outcomes, and each outcome i has a probability p_i.

$$E(I(X))=\sum_{i=1}^{N} p_i \cdot \log_2\left(\frac{1}{p_i}\right)$$

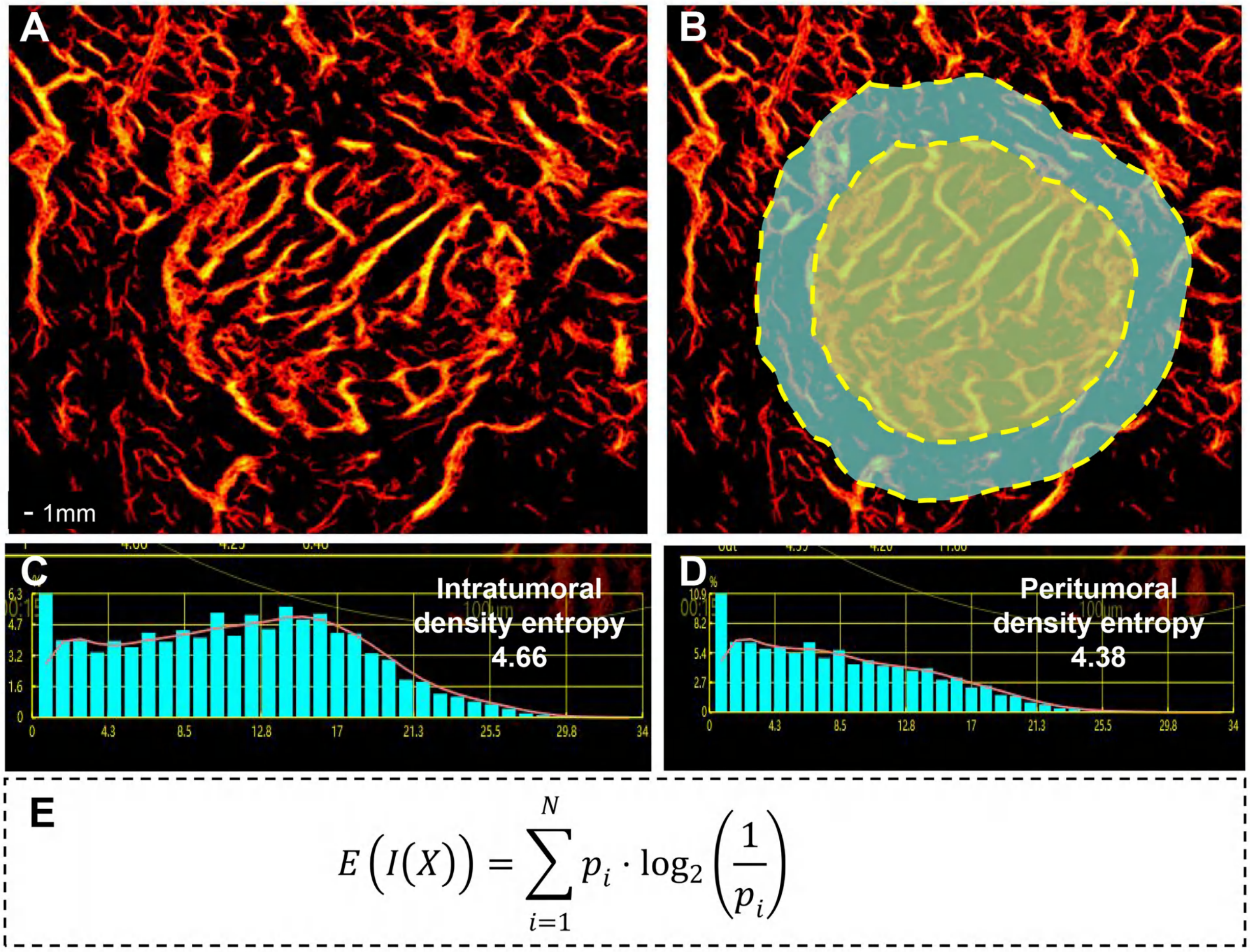

Figure S7. Calculation method for density entropy. (A) Density map; (B) Intratumoral (yellow) and peritumoral 0.5cm (blue) ROIs were depicted. (C-E) The density entropy of intratumoral and peritumoral ROIs are calculated using the entropy formula by substituting the probability p_i for each interval. ROI, region of interest. Scale bar: 1 mm.

(3) Vessel ratio

Based on the spatial distribution in the density map, the vessel ratio is obtained by calculating the proportion of vascular area within the ROIs after extracting vascular structures via binarization (**Figure S8**).

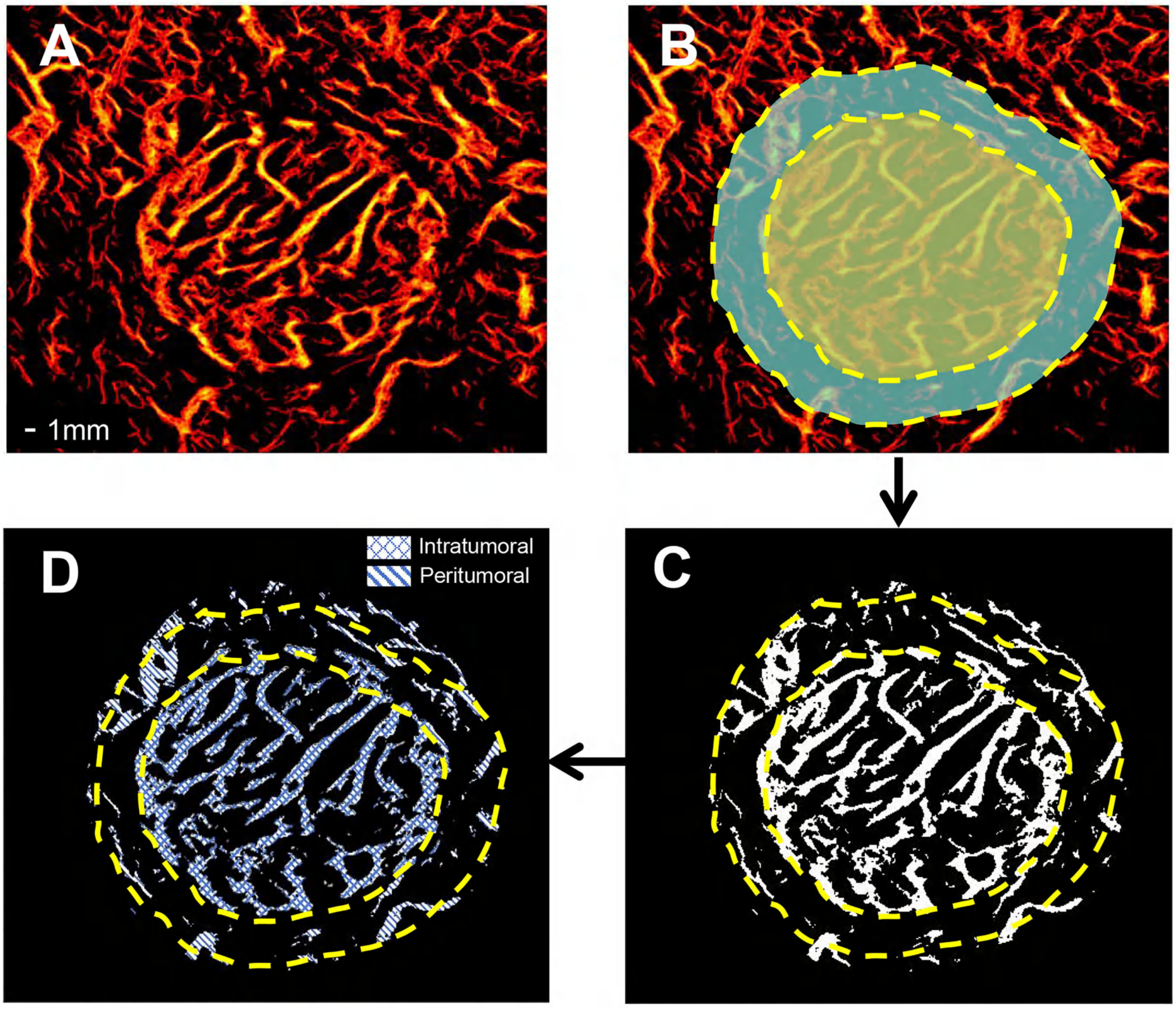

Figure S8. Calculation method for vessel ratio. (A) Density map; (B) Intratumoral (yellow) and peritumoral 0.5cm (blue) ROIs were depicted; (C) Microvascular structures extracted using binary segmentation in ROI; (D) Pixel counting in the microvascular area. ROI, region of interest. Scale bar: 1 mm.

(4) Complexity level

The complexity level is quantified by calculating the fractal dimension of the vascular structures. It is defined as the slope of fitting curve between the number of boxes covering the vessels and the box sizes (**Figure S9**).

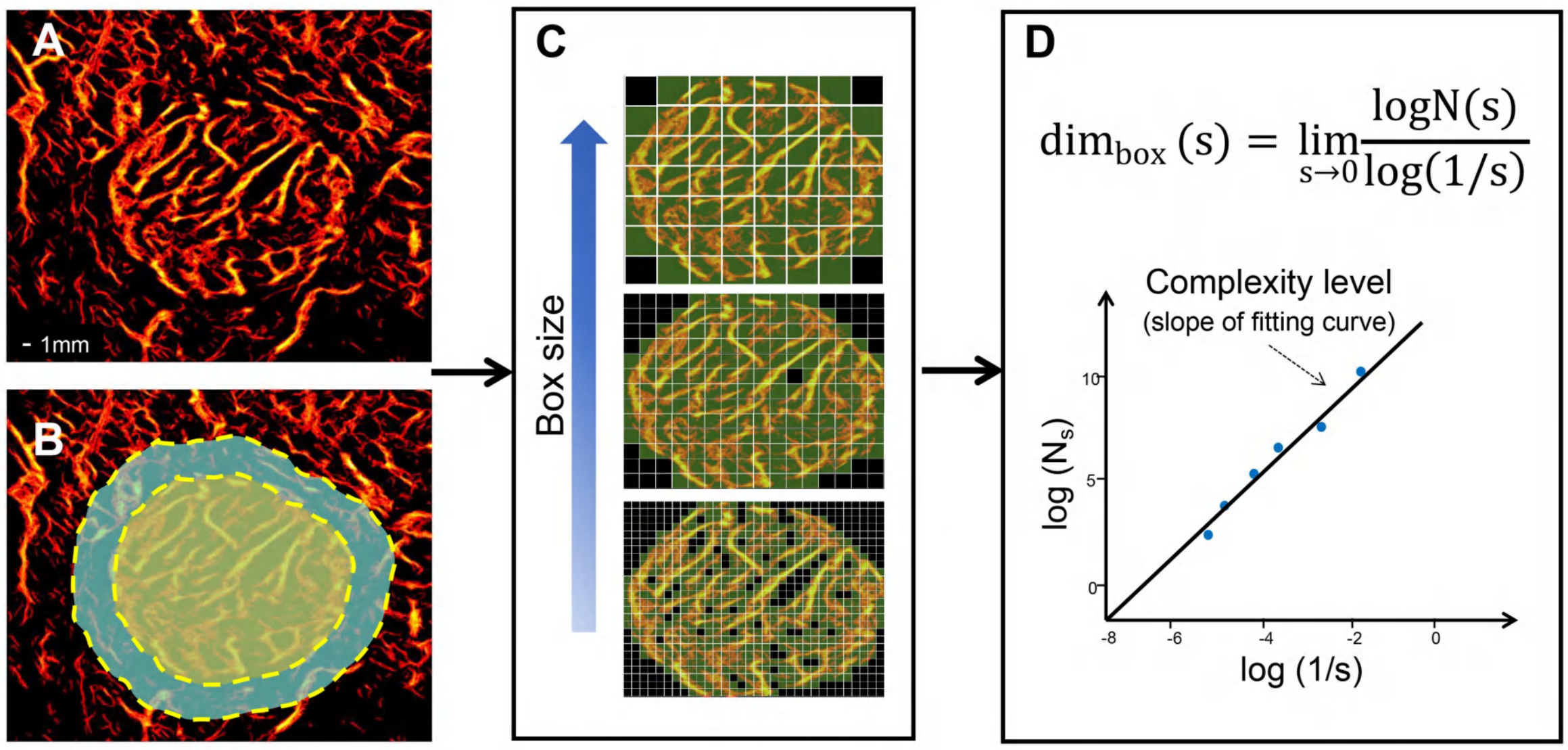

Figure S9. Calculation method for complexity level. (A) Density map; (B) Intratumoral (yellow) and peritumoral 0.5cm (blue) ROIs were depicted; (C) The microvessel density map is partitioned with progressively smaller grids, and the number of boxes containing vessels is counted; (D) By covering the vessel image with boxes of different sizes and counting the number of boxes containing vessels (N(s)), the relationship between the logarithm of the box count (logN(s)) and the logarithm of the inverse of the box size (log(1/s)) is analyzed on a logarithmic scale. The slope of this relationship represents the complexity level. ROI, region of interest. Scale bar: 1 mm.

(5) Velocity parameters (maximum velocity, minimum velocity, mean velocity, standard deviation of velocity)

Velocity parameters represent the distance that fluid travels within a vessel per unit time. The greater the distance traveled per unit time, the faster the vascular velocity. The quantitative parameters related to vascular velocity are calculated as follows (**Figure S10**).

1) Max velocity = the maximum vascular velocity within the ROI.

2) Min velocity = the minimum vascular velocity among regions with velocity greater than zero within the ROI.

3) Mean velocity (MeanVel) = the sum of vascular velocities in regions with velocity greater than zero within the ROI, divided by the number of pixels in those regions.

$$\text{MeanVel} = \frac{\sum_{i \in \{\text{ voxels with } v_i>0\}} v_i}{n_{vel>0}}$$

($n_{vel} > 0$ is the number of voxels with vascular velocity greater than zero in the ROI.)

4) Standard deviation of velocity (Std Vel) = the sample standard deviation of vascular velocity values over pixels with vascular velocity greater than zero in the ROI.

$$\text{Std Vel}=\sqrt{\frac{1}{n-1}\sum_{i=1}^{n}(\text{Vel}_i-\text{MeanVel})^2}$$

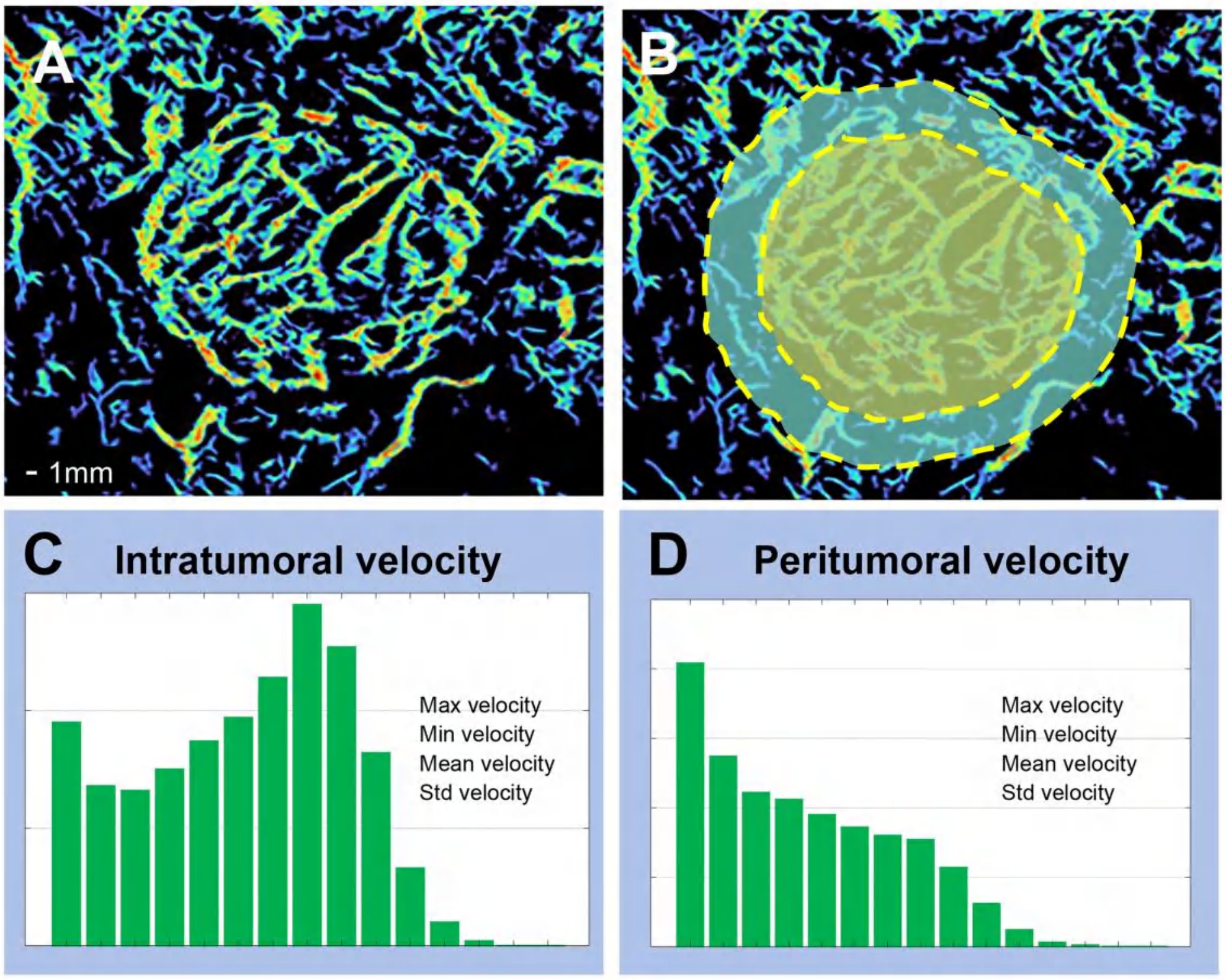

Figure S10. Calculation method for velocity parameters. (A) Velocity map; (B) Intratumoral (yellow) and peritumoral 0.5cm (blue) ROIs were depicted; (C-D) Max velocity, min velocity, mean velocity and Std velocity were calculated by analyzing numerical distribution of velocity in two ROIs. ROI, region of interest. Std, standard deviation. Scale bar: 1 mm.

(6) Velocity entropy

Velocity entropy measures the uniformity of blood flow velocity variations within the microvessels. If the flow velocity varies significantly between different vessels, the entropy value is higher. The velocity values are grouped into intervals (e.g., slow, medium, fast), and the probability p_i for each interval is calculated (**Figure S11**).

Entropy is calculated using this formula, where X is a random variable with N possible outcomes, and each outcome i has a probability p_i.

$$E\left(I(X)\right) = \sum_{i=1}^{N} p_i \cdot \log_2\left(\frac{1}{p_i}\right)$$

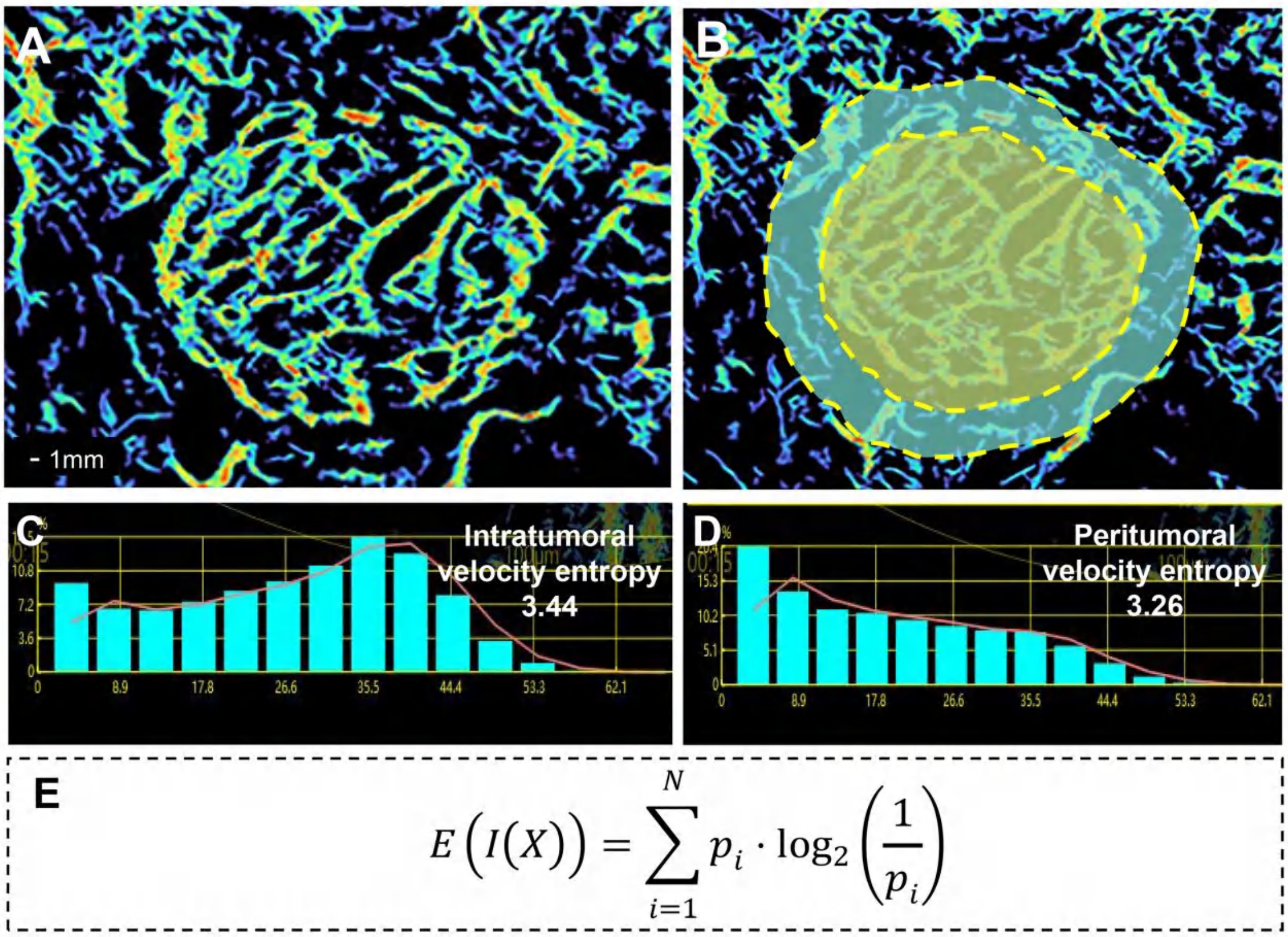

Figure S11. Calculation method for velocity entropy. (A) Velocity map; (B) Intratumoral (yellow) and peritumoral 0.5cm (blue) ROIs were depicted; (C-E) The velocity entropy of intratumoral and peritumoral ROIs are calculated using the entropy formula by substituting the probability p_i for each interval. ROI, region of interest. Scale bar: 1 mm.

(7) Curvature (SOAM)

Vascular curvature represents the degree of curvature of the vessel. The greater the vascular curvature value, the more curved the vessel course. Curvature SOAM is defined as an index for quantitative analysis of the local curvature characteristics of blood vessels (**Figure S12**). Using vascular connectivity, the start and end points of vessels within the ROI are automatically identified. The actual distance (A) and Euclidean distance (E) of each vessel are calculated.

The formula of calculating curvature (SOAM) is as follows. The vector angle θi is calculated for each group of three consecutive points of ai, ai+1 and ai+2, and the normalized angle between all three consecutive points of the whole blood vessel is added to obtain the SOAM value. ROI, region of interest; SOAM, sum of angles metric.

$$A = \sum_{i=1}^{N} a_i \qquad DM = \frac{A}{E} \qquad SOAM = \sum_{i=1}^{N-1} \frac{\theta_i}{a_i}$$

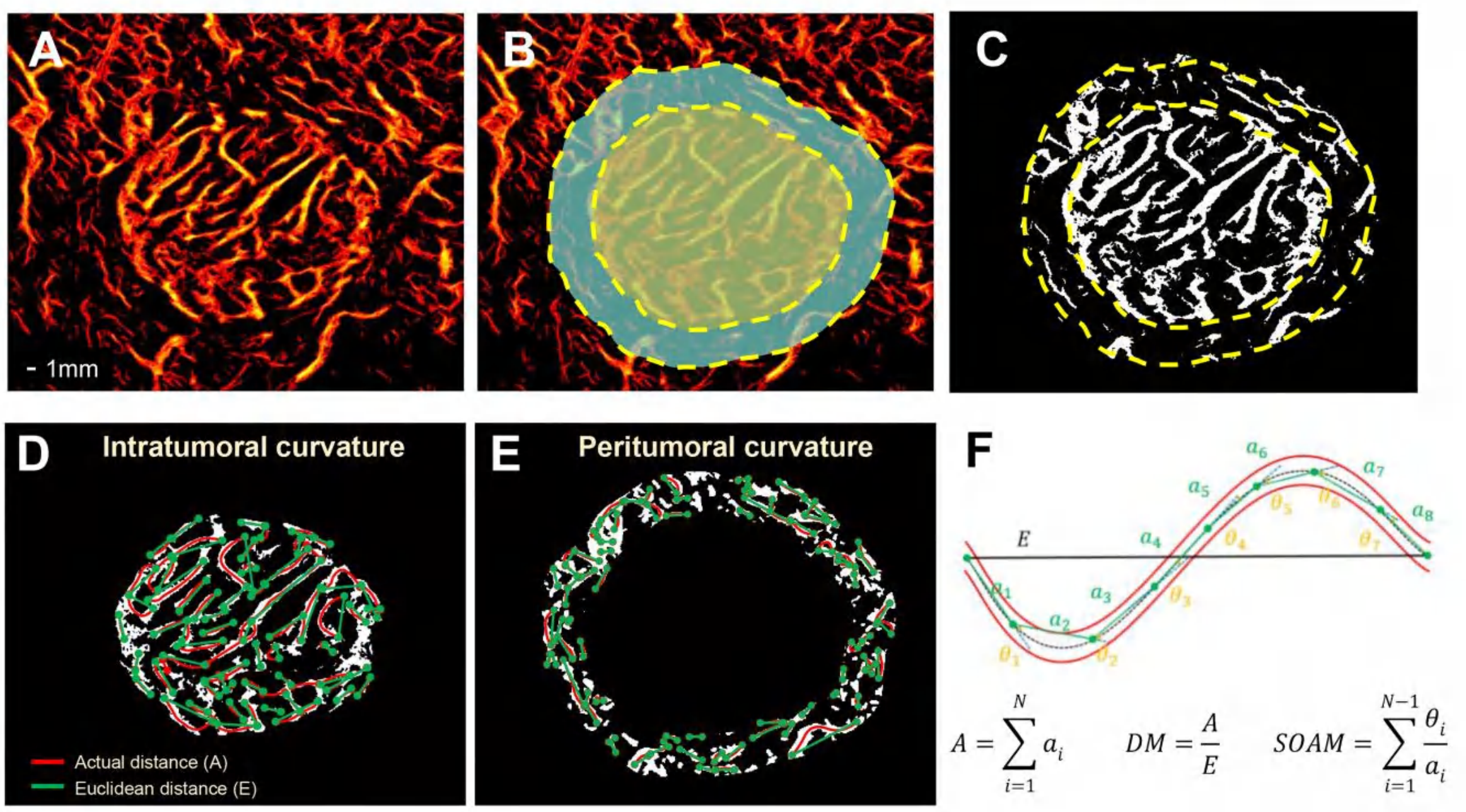

Figure S12. Calculation method for curvature (SOAM). (A) Density map; (B) Intratumoral (yellow) and peritumoral 0.5cm (blue) ROIs were depicted; (C) Microvascular structures extracted using binary segmentation in ROI; (D-E) Actual distance (red) and Euclideandistance (green) extracted using skeleton algorithm on the identified vessels; (F) The calculated formula of curvature (SOAM). ROI, region of interest; SOAM, sum of angles metric. Scale bar: 1 mm.

(8) Perfusion index

The perfusion index is then calculated by multiplying the mean velocity within the ROI by the vessel ratio.

$$\text{Perfusion index} = (\text{mean velocity of ROI}) \times (\text{vessel ratio of ROI})$$

(9) Blood volume

Blood volume is defined as the total number of microbubbles entering the ROI during the total length of collection. The unit of blood volume is au. 1au = 1×10^4 microbubbles.

Note S5. Sample size calculation for intraclass correlation coefficient (ICC)

The purpose of this study was to establish a standardized ULM protocol for HCC. An internal pilot study involving 10 HCC patients was conducted. Two radiologists (J.J.T and H.Z.L) independently analyzed the ULM images (the same count of reconstructed frames) and calculated the quantitative parameters within the ROIs. The intraclass correlation coefficient (ICC) for all ULM quantitative parameters was 0.932 (95% confidence interval [CI]: 0.892 to 0.973). To ensure a precise estimate of reliability in the formal study, we targeted a narrower CI for the ICC. Based on the pilot result (ICC = 0.932), a target 95% CI width of 0.05 was specified. Using PASS software (version 21.0.9), with parameters set to two raters, a two-sided confidence level of 95%, and the planned ICC of 0.932, the analysis indicated that a minimum of 30 patients is required to achieve the desired precision. Therefore, we plan to enroll 30 patients with HCC for the final consistency analysis phase of this standardized methodology study.

Note S6. Curve and group analysis of ULM structural-functional parameters with the increase of frame counts.

Group comparisons for all ULM structural-functional parameters are summarized in **Figure S13**. Hemodynamic parameters (vessel ratio, blood volume, perfusion index) and density parameters (maximum density, mean density, density entropy) differed significantly across the three frame groups (repeated-measures ANOVA, all $p < 0.001$). Post-hoc analyses revealed that these differences were primarily driven by the low-frame group, while no significant differences were detected between the medium- and high-frame groups for any of

the evaluated parameters. Velocity and curvature parameters showed no significant variation across frame counts (all $p \geq 0.05$).

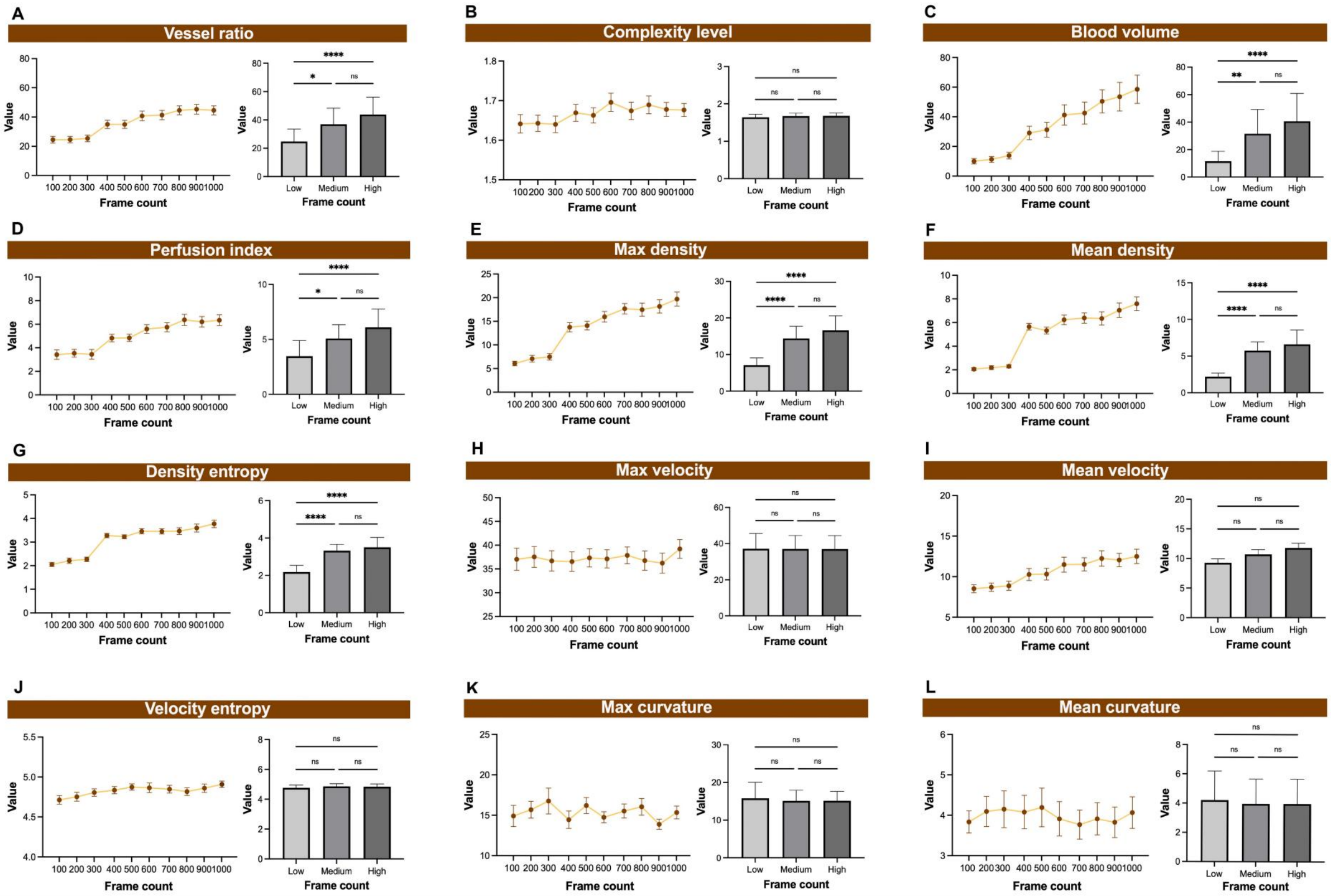

Figure S13. Curve and grouping analysis of ULM parameters with the increase of frame counts. (A) Hemodynamic parameters (vessel ratio, complexity level, blood volume and perfusion index) gradually increased as the frame count elevated. Only vessel ratio, blood volume and perfusion index displayed great difference between the low and high frame groups. (E-G) Density parameters (max density, mean density and density entropy) progressively rose as the frame count increased, and showed a significant increase when the frame counts exceeded 400. Three density parameters obviously elevated in both medium and high groups compared to the low group, with no difference between medium-high groups. (H-J) Max velocity had no obvious change, but mean velocity and velocity entropy showed slightly rose as frame count increased. Three velocity parameters showed no significant differences among different frame groups. (K, L) Curvature parameters (max velocity, mean velocity) displayed slightly decreased as frame increased. No significant difference was shown among different frame groups. Data are presented as mean ± standard deviation (n = 61). ns, $p \geq 0.05$; *, $p < 0.05$; **, $p < 0.01$; ****, $p < 0.0001$. ULM, ultrasound localization microscopy

Note S7. Comparison of CEUS metrics and ULM parameters in different HCC subtypes

Table S1. CEUS metrics in different HCC subtypes

Parameters	Differentiation grade			MVI status		
	p-HCC (n = 11)	w-HCC (n = 50)	*p* value	Positive (n = 30)	Negative (n = 31)	*p* value
AUC	5132.63 ± 2271.80	6175.60 ± 2501.21	0.209	5665.42 (4204, 6942.9)	5811.90 (4367.5, 8054.9)	0.868
Peak intensity	59.30 ± 17.39	68.91 ± 16.51	0.089	68.07 ± 17.84	66.30 ± 16.26	0.687
Time to peak	35.02 (28.89, 42.02)	31.47 (25.35, 38.98)	0.151	31.47 (25.88, 37.37)	31.96 (26.63, 39.81)	0.926
Arrival time	15.85 (12.62, 18.23)	13.14 (9.99, 14.96)	0.119	13.13 (10.14, 14.67)	13.65 (11.30, 17.02)	0.352
Rising time	16.80 (14.90, 21.12)	17.40 (14.31, 22.69)	0.859	16.76 (14.27, 19.72)	17.15 (14.89, 23.02)	0.891
MTT	108.41 ± 26.04	117.06 ± 30.23	0.383	117.68 ± 27.61	113.38 ± 31.54	0.573

Note: HCC, hepatocellular carcinoma; CEUS, contrast-enhanced ultrasound; MVI, microvascular invasion; AUC, area under curve. MTT, mean transit time.

Table S2. ULM parameters in different HCC subtypes

**Parameters**	**Differentiation grade**			**MVI status**		
	p-HCC (n = 11)	w-HCC (n = 50)	*p* value	Positive (n = 30)	Negative (n = 31)	*p* value
**Intratumoral**						
Vessel ratio	30.97 ± 13.77	37.85 ± 15.61	0.183	31.80 ± 15.06	41.25 ± 14.53	**0.015**
Complexity level	1.62 ± 0.08	1.66 ± 0.09	0.200	1.63 ± 0.08	1.67 ± 0.09	0.152
Blood volume	34.40 (19.46, 50.65)	19.78 (10.37, 52.79)	0.316	22.24 (12.71, 46.23)	20.11 (9.53, 54.59)	0.780
Perfusion index	4.09 (2.49, 6.16)	4.75 (2.89, 7.59)	0.334	4.03 (1.93, 5.79)	5.35 (4.04, 8.35)	**0.019**
Max density	17.93(14.46, 23.08)	15.29 (8.95, 18.40)	0.063	14.34 ± 6.74	15.34 ± 6.15	0.546
Mean density	7.61 (5.36, 8.44)	4.84 (2.67, 5.99)	**0.009**	4.33 (2.76, 5.76)	5.27 (3.02, 6.27)	0.267
Density entropy	3.78 ± 0.23	2.86 ± 0.65	**< 0.001**	2.88 ± 0.66	2.97 ± 0.60	0.590
Max velocity	38.95(36.13, 42.65)	37.94 (31.45, 42.85)	0.404	34.43 (30.16, 42.26)	39.84 (34.99, 43.31)	0.094

**Parameters**	**Differentiation grade**			**MVI status**		
	p-HCC (n = 11)	w-HCC (n = 50)	*p* value	Positive (n = 30)	Negative (n = 31)	*p* value
Mean velocity	9.62 ± 2.59	10.33 ± 3.73	0.556	9.24 ± 3.13	11.13 ± 3.73	**0.037**
Velocity entropy	4.56 (4.43, 4.85)	4.62 (4.36, 4.88)	0.881	4.54 ± 0.34	4.67 ± 0.34	0.129
Max curvature	15.29 (13.19, 16.91)	14.93 (12.78, 18.28)	0.851	15.97 (14.10, 18.35)	13.78 (12.19, 15.91)	0.164
Mean curvature	7.25 (6.59, 8.21)	3.06 (2.52, 3.93)	**< 0.001**	3.08 (2.32, 4.26)	2.77 (2.50, 3.38)	0.313
**Peritumoral**						
Vessel ratio	22.10 ± 10.88	24.66 ± 12.66	0.536	22.78 ± 12.22	25.57 ± 12.45	0.381
Complexity level	1.42 (1.34, 1.43)	1.45 (1.33, 1.51)	0.256	1.37 (1.29, 1.47)	1.47 (1.40, 1.51)	0.050
Blood volume	10.90 (8.51, 17.91)	10.84 (4.12, 16.89)	0.619	11.10 (5.09, 17.29)	10.90 (4.40, 16.77)	1.000
Perfusion index	2.79 (1.61, 3.23)	3.32 (1.53, 4.76)	0.476	2.43 (1.40, 4.68)	3.35 (2.27, 4.51)	0.226
Max density	18.99 ± 4.04	12.34 ± 4.85	**< 0.001**	13.81 ± 6.30	15.10 ± 6.34	0.428
Mean density	4.61 (3.84, 6.24)	4.28 (2.38, 5.34)	0.186	3.91 (2.45, 5.11)	4.41 (2.97, 5.53)	0.385
Density entropy	3.03 ± 0.50	2.83 ± 0.66	0.348	2.81± 0.65	2.92 ± 0.63	0.486
Max velocity	36.00 (30.89, 39.22)	35.33 (30.09, 42.19)	0.873	33.52 (29.98, 39.43)	36.81 (30.20, 42.80)	0.215
Mean velocity	8.28 (7.27, 10.09)	8.96 (6.49, 10.92)	0.859	8.33 ± 2.67	9.59 ± 3.41	0.115
Velocity entropy	4.61 ± 0.24	4.56 ± 0.40	0.741	4.51 ± 0.34	4.63 ± 0.39	0.178
Max curvature	10.54 (9.55, 11.25)	12.69 (10.50, 15.15)	0.079	12.52 (10.37, 14.89)	12.11 (10.36, 14.87)	0.747
Mean curvature	2.25 (2.02, 2.6)	2.53 (2.10, 3.08)	0.171	2.73 (2.26, 3.70)	2.18 (1.97, 2.66)	**0.003**

Note: ULM, ultrasound localization microscopy; HCC, hepatocellular carcinoma; p-HCC, poorly-differentiated HCC; w-HCC, well-differentiated HCC; MVI, microvascular invasion.

Note S8. Sensitivity analysis with 1:2 random subsampling of w-HCC cases

To evaluate the impact of sample imbalance on the robustness of our findings, we performed a sensitivity analysis using a 1:2 random subsampling approach. From the 50 w-HCC cases enrolled during the same period as the p-HCC group, 22 were randomly selected using a computer-generated random number sequence (1:2 ratio). The randomly selected w-HCC subset showed no significant differences in baseline characteristics and CEUS quantitative metrics compared with the p-HCC group (**Tables S3 and S4**). Re-analysis of the ULM parameters demonstrated that intratumoral mean curvature remained an independent predictor for p-HCC (OR 3.57, 95% CI 1.19–10.73, $p = 0.023$) (**Tables S5 and S6**). The diagnostic sensitivity, specificity, accuracy, and AUC of intratumoral mean curvature were 90.9%, 95.5%, 93.9%, and 0.98 (95% CI 0.93–0.99), respectively. These results were highly consistent with the original analysis, confirming that the group size imbalance did not materially affect our primary conclusions.

Table S3. Characteristics of HCC differentiation grade groups (n = 33)

**Characteristics**	**Differentiation grade**		
	p-HCC (n = 11)	w-HCC (n = 22)	*p* value
**Age (years)**	57.18 ± 7.31	60.05 ± 8.63	0.353
**Gender**			1.000
Male	10 (90.9%)	19 (86.4%)	
Female	1 (9.1%)	3 (13.6%)	
**Tumor size (cm)**	4.90 (4.20, 6.60)	4.25 (3.08, 5.93)	0.339
**Cirrosis**	5 (45.5%)	4 (18.2%)	0.121
**HBsAg positive**	10 (90.9%)	14 (63.6%)	0.212
**HBsAb positive**	1 (9.1%)	3 (13.6%)	1.000
**HBeAg positive**	5 (45.5%)	3 (13.6%)	0.082
**HBeAb positive**	5 (45.5%)	10 (45.5%)	1.000
**HBcAb positive**	10 (90.9%)	16 (72.7%)	0.378
**Laboratory values**			
AFP ≥ 20 ng/mL	7 (63.6%)	10 (45.5%)	0.465
NLR	2.33 ± 1.14	2.34 ± 1.13	0.974
PLR	100.00 (74.59, 145.74)	95.23 (84.30, 121.85)	0.836

Note: HCC, hepatocellular carcinoma; p-HCC: poorly-differentiated HCC; w-HCC: well-differentiated HCC; AFP, α-fetoprotein; HBsAg, Hepatitis B surface antigen; HBsAb, Hepatitis B surface antibody; HBeAg, Hepatitis B e antigen; HBeAb, Hepatitis B e antibody; HBcAb, Hepatitis B core antibody; NLR = neutrophil count (×10^9/L) / lymphocyte count (×10^9/L). PLR = platelet count (×10^9/L) / lymphocyte count (×10^9/L)

Table S4. CEUS metrics in HCC differentiation grade groups (n = 33)

Parameters	Differentiation grade		
	p-HCC (n = 11)	w-HCC (n = 22)	*p* value
AUC	5132.60 ± 2271.80	4998.50 ± 1783.00	0.854
Peak intensity	59.30 ± 17.39	64.61 ± 14.45	0.359
Arrival time	15.85 (12.62, 18.23)	13.44 (9.99, 14.72)	0.122
Rising time	16.80 (14.90, 21.12)	16.64 (14.34, 18.93)	0.611
MTT	105.54 (102.37, 125.70)	105.72 (102.93, 109.17)	0.534

Note: HCC, hepatocellular carcinoma; p-HCC, poorly-differentiated HCC; w-HCC, well-differentiated HCC. CEUS, contrast-enhanced ultrasound; AUC, area under curve. MTT, mean transit time.

Table S5. ULM quantitative parameters in HCC differentiation grade groups (n = 33)

Parameters	Differentiation grade		
	p-HCC (n = 11)	w-HCC (n = 22)	*p* value
**Intratumoral**			
Vessel ratio	30.97 ± 13.77	31.89 ± 13.91	0.858
Complexity level	1.62 ± 0.08	1.63 ± 0.09	0.671
Blood volume	34.40 (19.46, 50.65)	19.39 (10.13, 38.04)	0.166
Perfusion index	4.16 ± 2.27	3.81 ± 2.14	0.664
Max density	18.38 ± 6.67	13.05 ± 5.65	**0.022**
Mean density	6.79 ± 1.89	3.86 ± 1.81	**< 0.001**
Density entropy	3.78 ± 0.23	2.66 ± 0.60	**< 0.001**
Max velocity	39.64 ± 5.39	34.05 ± 5.10	**0.007**
Mean velocity	9.62 ± 2.59	8.29 ± 2.38	0.150
Velocity entropy	4.61 ± 0.26	4.44 ± 0.27	0.093
Max curvature	15.08 ± 2.60	15.44 ± 3.39	0.765
Mean curvature	7.04 ± 2.20	2.90 ± 0.52	**< 0.001**

Parameters	Differentiation grade		
	p-HCC (n = 11)	w-HCC (n = 22)	*p* value
**Peritumoral**			
Vessel ratio	22.10 ± 10.88	23.13 ± 13.25	0.825
Complexity level	1.38 ± 0.12	1.40 ± 0.16	0.759
Blood volume	10.90 (8.51, 17.91)	10.21 (4.12, 16.02)	0.440
Perfusion index	2.79 (1.61, 3.23)	2.70 (1.08, 3.73)	0.834
Max density	18.99 ± 4.04	16.20 ± 3.66	0.412
Mean density	5.18 ± 2.14	3.55 ± 1.69	**0.023**
Density entropy	3.03 ± 0.50	2.66 ± 0.62	0.095
Max velocity	35.37 ± 5.62	31.72 ± 5.52	0.085
Mean velocity	8.28 (7.27, 10.09)	7.77 (5.84, 9.10)	0.141
Velocity entropy	4.61 ± 0.24	4.36 ± 0.30	**0.028**
Max curvature	11.12 ± 3.58	13.23 ± 5.16	0.236
Mean curvature	2.25 (2.02, 2.60)	2.40 (2.10, 2.72)	0.674

Note: ULM, ultrasound localization microscopy; HCC, hepatocellular carcinoma; p-HCC, poorly-differentiated HCC; w-HCC, well-differentiated HCC; MVI, microvascular invasion.

Table S6. Univariate and multivariate logistic regression analyses of ULM parameters for predicting poorly-differentiated HCC (n = 33).

Parameters	Univariate analysis		Multivariate analysis	
	Odds ratio (95% CI)	*p* value	Odds ratio (95% CI)	*p* value
Intratumoral max density	1.09 (0.95 - 1.24)	0.221	-	-
Intratumoral mean density	2.19 (1.29 - 3.71)	**0.004**	1.82 (0.71 - 4.66)	0.215
Intratumoral mean curvature	5.00 (1.58 - 15.81)	**0.006**	3.57 (1.19 - 10.73)	**0.023**
Peritumoral mean density	1.38 (0.90 - 2.10)	0.140	-	-
Peritumoral velocity entropy	4.57 (0.38 - 55.55)	0.233	-	-

Note: ULM, ultrasound localization microscopy; HCC, hepatocellular carcinoma. CI, confidential interval.

Note S9. Comparison of ULM reconstruction between peak-intensity and wash-out phases in HCC patients.

We enrolled five HCC patients and acquired ULM data at two phases during the same contrast injection: the peak-intensity phase (identified by the TIC) and the wash-out phase (delayed period with sparse microbubbles, 2 minutes after injection). ULM reconstructions were generated from the two phases using the identical processing method described in the main text. ULM resolution indices and structural-functional parameters were extracted and compared from each reconstruction.

The results showed that peak-intensity ULM yielded significantly higher vessel counts ($p = 0.047$), vessel ratio ($p = 0.027$), perfusion index ($p = 0.003$), and mean density ($p = 0.012$) compared with ULM reconstruction in the wash-out phase. Other parameters showed no statistically significant differences between the two phases (all $p \geq 0.05$) (**Figure S14**).

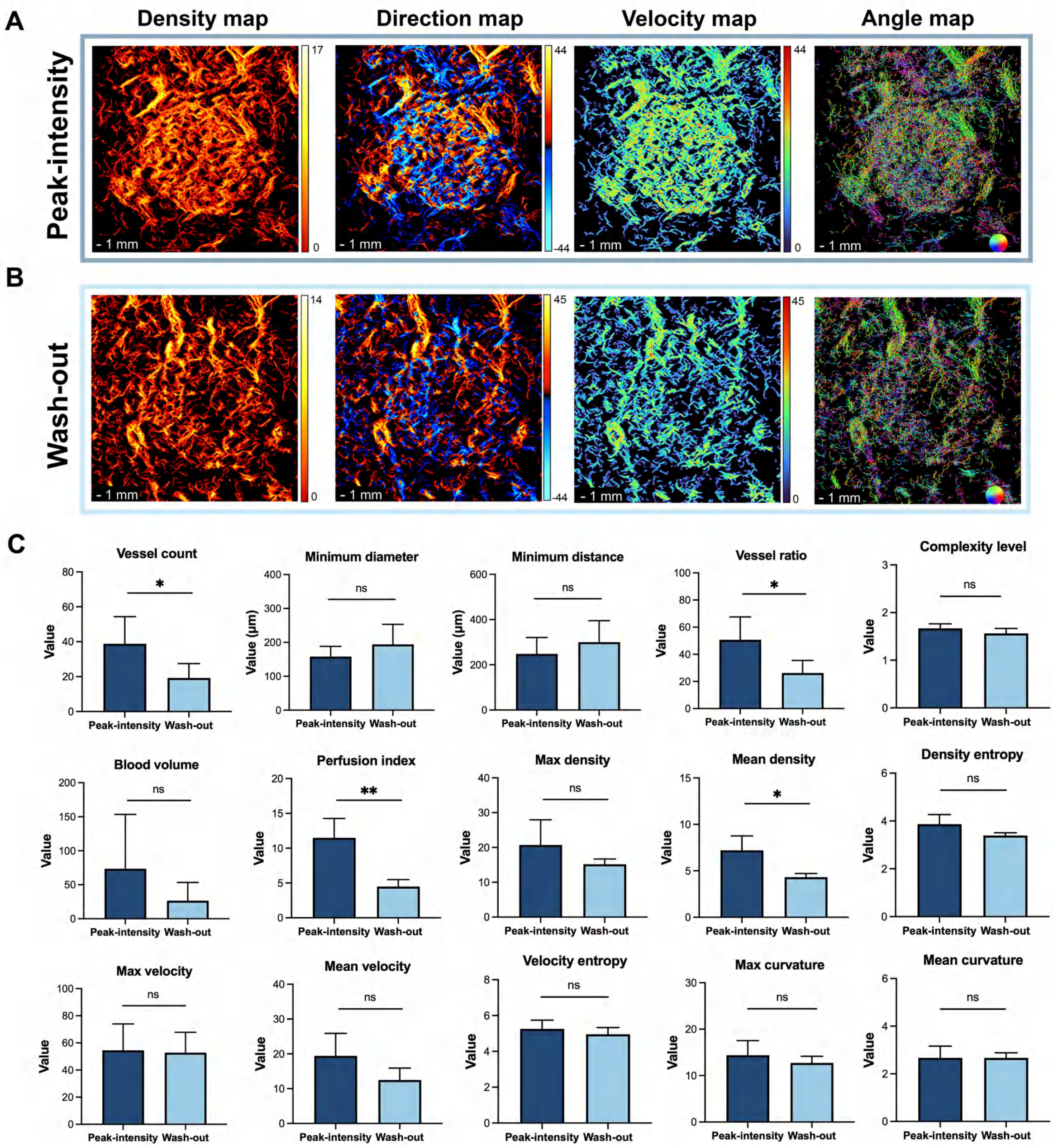

Figure S14. Comparison of ULM reconstruction between peak-intensity and wash-out phases in HCC patients. (A) Representative ULM maps reconstructed from the peak-intensity phase. (B) Corresponding ULM images reconstructed from the wash-out phase of the same patient. (C) Bar charts (mean ± standard deviation, n = 5) comparing ULM parameters. ns， $p \geq 0.05$, * $p < 0.05$, ** $p < 0.01$. Scale bar: 1 mm.

Note S10: Comparison of ULM reconstruction between 1000 frames and 3000 frames in HCC patients.

Three HCC patients capable of a 20-second breath-hold (motion variance $\leq$ 10%) underwent continuous 3000-frame acquisition at 150 Hz using a convex probe. The full 3000-frame dataset and the first 1000 frames were processed with the ULM protocol, from which ULM resolution indices and ULM structural–functional parameters were extracted. Intraclass correlation coefficients (ICC) were calculated for each parameter to quantify consistency between two groups.

ULM resolution indices showed good to excellent agreement between 1000-frame and 3000-frame reconstructions (ICC: 0.852–0.943), with no significant difference (all $p \geq 0.05$). Hemodynamic parameters also exhibited high consistency (ICC: 0.764–0.982). The 3000-frame values were slightly higher but neither statistically distinct (all $p \geq 0.05$). Curvature parameters were highly consistent (ICC: 0.873–0.882) and showed negligible group differences (all $p \geq 0.05$). By contrast, density and velocity parameters were systematically higher in the 3000-frame group and had uniformly low ICCs ($\leq$ 0.5). These differences did not reach statistical significance, likely due to the small sample size (**Figure S15**).

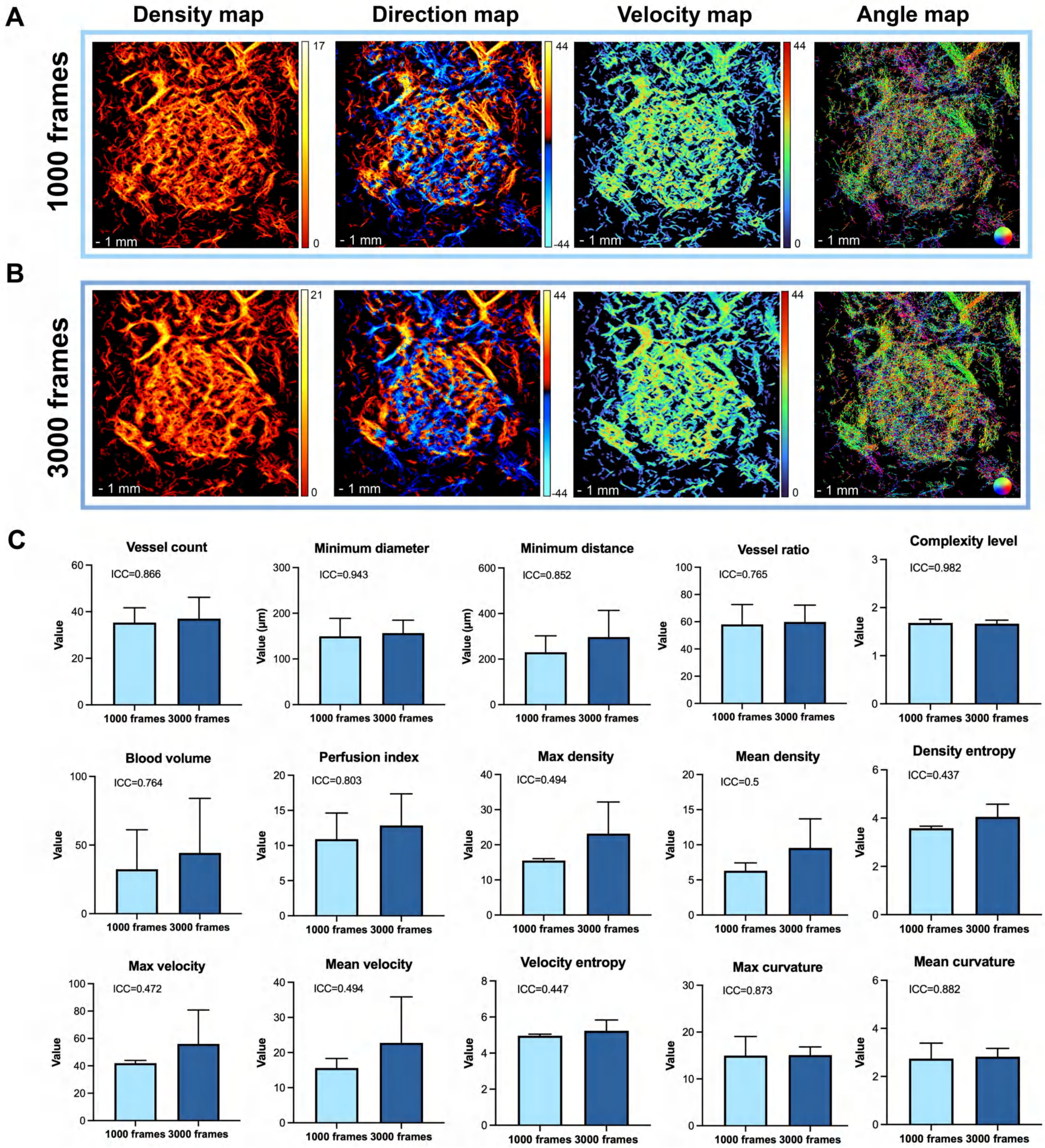

Figure S15. **Comparison of ULM reconstruction between 1000 frames and 3000 frames.** (A) Representative ULM image reconstructed from 1000 frames in a patient with HCC. (B) Corresponding ULM image reconstructed from the full 3000-frame acquisition of the same patient. (C) Bar charts (mean ± standard deviation, n = 3) comparing ULM parameters. Intraclass correlation coefficient (ICC) values are displayed above each parameter group. Scale bar: 1 mm.
